# Functioning Problems Associated with Health Conditions with Greatest Disease Burden in South Africa: A Scoping Review

**DOI:** 10.3390/ijerph192315636

**Published:** 2022-11-24

**Authors:** Maria Y. Charumbira, Karina Berner, Quinette A. Louw

**Affiliations:** Division of Physiotherapy, Department of Health and Rehabilitation Sciences, Faculty of Medicine and Health Sciences, Stellenbosch University, Cape Town 7500, South Africa

**Keywords:** functioning, ICF, primary care, rehabilitation, South Africa

## Abstract

A notable rise in health-related disability for which evidence-based rehabilitation is beneficial is evident in low-to-middle income countries. This scoping review aimed to systematically identify and map the most common functioning problems associated with health conditions that contribute most to disability in South Africa using the International Classification of Functioning, Disability and Health (ICF) framework. Peer-reviewed evidence published from January 2006 to December 2021 was systematically searched from five databases. Some 268 studies reporting on functioning problems (impairments, activity limitations, and participation restrictions) in South African adults (>18 years) related to 10 health conditions were included. A total of 130 different functioning problems were mapped to the ICF. The most prevalent problems (top 20) were related to mobility, pain, and mental health but spanned across several ICF domains and were mostly in patients at primary care. The high prevalence and wide range of functioning problems may be particularly burdensome on an already strained primary health care (PHC) system. This points towards targeted planning of innovative strategies towards strengthening rehabilitation service delivery at primary care to address these complexities where there is an inadequate rehabilitation workforce.

## 1. Introduction

Functioning of individuals is recognised as an important indicator of population health and well-being [1]. Functioning refers to the ability to perform daily activities and participation in personal, work and community settings [2]. Ageing, trauma and diseases, especially non-communicable chronic diseases (NCDs) impairs functioning [3], in the short-term, long-term, or episodically. Suboptimal functioning influences an individual’s perception of their health condition negatively [4]. Therefore, functioning problems compound the effect on perception of health and quality of life in people who are already living with morbidity associated with a chronic condition [4].

Morbidity at a national level is often described using Global Burden of Disease (GBD) metrics such as Years Lived with Disability (YLDs) [5]. The YLDs reflect the total years lived with a chronic condition disease [6]. YLDs are increasing at a higher rate in low- and middle- income countries (LMICs), compared to high- income countries (HICs) [7]. In South Africa (SA), YLDs due to health conditions that contribute to most disability increased by about 270% from 1990–2017, with a further 6% predicted increase from 2017–2022 [8]. Thus, health systems in LMICs need to adapt in response to the epidemiological transitioning reflective of high levels of morbidity and functioning problems [9].

To prepare countries, especially LMICs, the WHO launched initiatives to strengthen rehabilitation as the key health strategy aimed at addressing functioning problems [10]. These WHO initiatives include a package of evidence-based rehabilitation interventions that should be prioritized for integration into the health systems [11]. Additionally, the WHO developed the Rehabilitation Competency Framework (RCF) which ensures that the rehabilitation workforce has the required competencies and is capable of addressing its population’s rehabilitation needs [12]. Since the healthcare experiences and rehabilitation needs of the populations vary widely between countries, country specific functioning profiles are needed to effectively plan and integrate rehabilitation services into local health systems.

Strengthening rehabilitation into the health system of LMICs is challenging. As a case example, South Africa, classified as a upper-middle-income country, has a high disease burden inclusive of communicable and non-communicable diseases [13]. The health system is already constrained and fragmented. Two decades post-Apartheid, racial and political divides continue to affect the quality and access to healthcare by different population groups. The private health sector provides healthcare services to 16% of South Africa’s mostly affluent population. The remaining 84% of South Africa’s poorer population receives healthcare from the public sector which uses 48% of the allocated national healthcare budget [14]. The limited public sector resources has to fund many competing health needs considering the high burden of disease. Furthermore, one in five adult South Africans present with multimorbidity [15] and associated functioning problems are notable but rarely addressed, especially at primary care level where only 6% of the total rehabilitation workforce work [16]. Thus, integrating rehabilitation services will require strong advocacy supported by relevant data on functioning needs to inform service planning.

South Africa is currently reforming towards a National Health Insurance (NHI) policy [17]. The policy promises that disability and rehabilitation services will be “fully integrated into primary health care (PHC) with a view to increasing care, treatment and rehabilitation” [18]. An important first step was to include rehabilitation in the country’s PHC standard treatment guidelines (STGs), which are the crux of packages of care [19]. Due to competing demands on the limited healthcare budget in South Africa, crucial information regarding the functioning problems is required to guide policy makers in prioritizing, costing, and designing rehabilitation services fit for the local population. Since many stakeholders are involved in the NHI planning and implementation process, standardisation of terminologies are important [2].

The International Classification of Functioning, Disability, and Health (ICF) provides standard terminology for describing functioning [2]. This framework can be used to indicate functioning problems that include body impairments, activity limitations or participation restrictions that result from an individual with a health condition interacting with contextual factors such as environmental and personal factors [2]. The focus is removed from the health condition an individual presents with to what they have difficulty doing—for example, being more concerned with whether a person has difficulty walking rather than whether the person has HIV or stroke. The ICF is aetiologically neutral meaning and serves to classify data on functioning across health conditions [2].

Comprehensive comparable data on functioning across health conditions in LMICs, particularly South Africa, is scarce. The growing body of literature that applies the minimal generic ICF set [20] (a minimum generic set of ICF domains suitable for describing functioning) or the ICF Rehabilitation Core Set [21] is largely based in high income countries (HICs). The findings may not be generalizable to South Africa’s context with limited access to quality healthcare services [22] and different profiles of chronic conditions contributing to most disability [7]. Instruments used to capture disability statistics during South Africa population surveys or censuses, such as the Washington Group Short Set on Functioning (WG-SS) [23], do not include all critical ICF domains. This may result in under-identifying or underreporting of functioning problems. The World Bank Model Disability Survey (MDS), while more comprehensive, is quite new and to date has not been implemented in South Africa [24]. Country-level planning is often based on the GBD studies estimates of disability at country and global levels [24]. However, a gap exists in detailed descriptions of the functioning problems required to plan rehabilitation services.

Clinical data on functioning in South African adult populations is available but is siloed into the different health conditions or specific functioning problem [25]. A composite and comprehensive mapping of this data compared across health conditions will provide country level data on functioning which is useful for rehabilitation service planning [26]. The main aim of this review was to summarize and synthesize the status of peer-reviewed literature regarding the most common functioning problems presenting in the adult population in South Africa. This was done by describing the most prevalence types of functioning problems (impairments, activity limitations and participation restrictions). These functioning problems were associated with the top 10 conditions contributing most to years lived with disability in South Africa and for which evidence-based rehabilitation interventions to address associated disability exist [8]. The most prevalent functioning problems were mapped to the ICF framework.

## 2. Materials and Methods

A scoping review of the current evidence regarding functioning problems associated with the priority conditions in South Africa was conducted according to a predefined protocol [27], in line with the methodological framework developed by Arksey and O’Malley [28]. Corresponding guidance contained in the Joanna Briggs Institute Reviewers’ Manual was also considered [29]. The ICF framework guided our review to allow a standardised analysis of the identified impairments, activity limitations, and participation restrictions associated with priority conditions in South Africa, to determine which were most common. We reported the scoping review according to the Preferred Reporting Items for Systematic reviews and Meta-Analyses Extension for Scoping Reviews (PRISMA-ScR) guidelines (Supplementary File S1) [30].

The main research questions for this review were: (i) “What is the spectrum of functioning problems associated with conditions contributing most to YLDs in South Africa?”; (ii) “Which are the ICF domains and categories dominantly affected by the most prevalent functioning problems in adult South Africans?”

Eligibility of the research questions was informed by the Population, Exposure, Context, Outcome design (PECOd) framework [29]. This included the population (P) of patients >18 years with exposure (E) to at least one of the conditions contributing to the greatest YLD, as indicated by GBD 2019 data, within the context (C) of South Africa [8]. The outcomes (O) were the functioning problems investigated using all peer-reviewed study designs (D).

### 2.1. Search Method

The bibliographic databases of PubMed/MEDLINE, SCOPUS, Web of Science, EbscoHost (CINAHL and Africawide Information), and SABINET were searched for articles published from 1 January 2006 to 31 December 2021. This date range was selected to focus on current functioning problems, considering that constant improvements in clinical care, public health and technology may affect rates of disability [31]. A re-run of the searches was conducted in June 2022 to complete search of new studies that may have been published since the previous searches run in August 2021. Studies were considered eligible if full texts were available, reporting information regarding type and/or prevalence of the impairments, activity limitations or participation restrictions associated with the health condition. The languages of publication were restricted to English and Afrikaans, the most common languages for scholarly communication in South Africa.

Grey literature, including theses and dissertations, were not reviewed as we were looking for complete, peer-reviewed published data only. We excluded impairments which were not indicated for rehabilitation, for example, neck stiffness in Tuberculous meningitis or internal impairments such as vomiting. We excluded health related quality of life assessments as they focus on the individual’s values and expectations following disease or injury [32] rather than functioning problems in terms of impairments, activity limitations and participation restrictions.

The search strategies were drafted and refined with the assistance of a librarian from Stellenbosch University. An initial search was conducted in PubMed. Key search terms regarding functioning including “activity limitation”, “functional impairment”, “functional loss”, “disability”, and “participation restriction” were used in varying combinations with the search terms for the listed health conditions. After analysing text words contained in titles and abstracts, and index words selected to describe key articles, additional search terms were added to the search strategy. Similar searches of the remaining electronic databases were done by adapting the various combinations of identified key words to the unique searching features of the databases. We used a wide search to include Title, Abstract and Key Words fields, to avoid missing important articles whose titles may not reflect the content of the article. The final search strategies for the five databases are provided (Supplementary File S2). We manually reviewed the reference lists of eligible studies for studies that may have been missed during the initial database searches.

All database search results were transferred to Rayyan reference management software [33]. Deduplication of all retrieved articles was done in Rayyan prior to the initial phase of screening by title and abstract.

### 2.2. Study Selection

After deduplication, one reviewer (MYC) reviewed all the titles and abstracts using pre-determined criteria and consulted a second reviewer (KB) or third reviewer (QAL) when unsure. One reviewer (MYC) further reviewed the full texts of the eligible articles to check that they had the required information. The reasons for excluding ineligible studies after reviewing the full texts were documented. There was no need to contact any authors of the included studies for further data clarification or additional information during the eligibility assessment as all data were clear.

### 2.3. Charting the Data

To ensure consistency and clarity of charted data, the reviewers held discussions to determine the variables, and nature and extent of the information to be extracted from the eligible studies. A custom data extraction form (available online: http://osf.10/7h6xz accessed on 15 March 2022) was initially developed in Microsoft Excel and revised iteratively after being piloted on a sample of 33 full text articles on stroke. The data extraction process was however cumbersome and time-consuming as it involved clicking across several columns in Excel and having to copy and paste every data entry. The potential for several errors and omissions during data entry and ICF classification existed, which would have affected the validity of our results [34]. Additionally, we encountered difficulty in filtering the layers of information—several health conditions needed to be considered to get an overview of functioning problems compared across health conditions, and each health condition was often associated with several functioning problems. These practical difficulties and concerns led to the design and development of a secure, web-based software application named *Rehab4all* [35]. One reviewer (MYC) subsequently extracted data from all eligible studies using the *Rehab4all* application while a second reviewer (QAL) checked for accuracy.

Data items included the first author and year of publication, study design, setting (rural, urban, or semi-urban), level of care (rehabilitation centre, PHC facility, specialized hospital), health condition (using the International Classification of Diseases 11th revision: ICD-11 [36] in combination with the GBD Institute for Health Metrics [37]), multimorbidity (the co-existence of two or more chronic health conditions in an individual), study design and description of sample population, including sex, and mean age. Details regarding outcome measures used to evaluate function, and the type and prevalence of functioning problems as reported in the articles, were recorded. Where several similar words or synonyms were found to report the same presenting functioning problem, the most familiar word in the South African layman language was selected—for example, “fatigue” and “exhaustion” were represented by “tiredness”. The presenting functioning problems were categorised into major groups based on similar functioning as listed in the Box 1 below.

Box 1 **The presenting functioning problems were categorized into major groups based on similar functioning.**

Balance and coordination problems Behaviour
problems Bowel and bladder problems Communication
problems Fine motor skill problems IADLs problems Joint
mobility/stiffness Mental disorders Mobility problems Muscle
function/paralysis Pain (acute, chronic, unspecified)Physical capacity problems Respiratory
problems Self-care problems Sensory problems Skin
problems Swallowing problems Swelling Vision
problems Weight problems Working and schooling
problems Sexuality problems

Prevalence statistics reporting on different recall periods (point, annual or lifetime) of the functioning problems were extracted. In longitudinal studies, where both baseline and post-intervention prevalence were reported, the baseline was considered as this is when the patient potentially begins to receive rehabilitation. Even for conditions like fractures or Tuberculosis, where rehabilitation is started after medical interventions, baseline data were considered as this time point present the worst-case scenario required for strategic planning [38]. There was no need to contact any authors for further details regarding unclear or incomplete data.

### 2.4. Quality Assessment

We did not appraise methodological quality or risk of bias of the included studies, in line with guidance on scoping review methodology [29].

### 2.5. Data Analysis

#### 2.5.1. Type of Functioning Problems

One reviewer (MYC) deductively coded the presenting functioning problems reported in the included studies, using the ICF framework with assistance of the *Rehab4all* application. The first-level classifications in the ICF are coded with a letter referring to the different components, where b = body functions (nine domains) and s = body structures (nine domains), and d = activities and participation (10 domains). The various components can be further coded with a number referring to second-level domains, third-level categories, and fourth-level qualifiers. Because of the use of the *Rehab4all* application, human error in coding was minimised. A second reviewer (QAL) checked for completeness and accuracy of the coding. Where the presenting functioning problem reported in the study could not be straightforwardly coded, the main concepts from the assessment tool or outcome measures used to evaluate functioning and vulnerability were used to derive the activity limitation or impairment using ICF linking rules [39].

#### 2.5.2. Prevalence of the Functioning Problems

The pooled prevalence of the identified impairments, activity limitations and/or participation restrictions were calculated. The top 20 most prevalent functioning problems were based on pooled prevalence if at least 5 articles provided prevalence statistics.

## 3. Results

**Figure 1:**
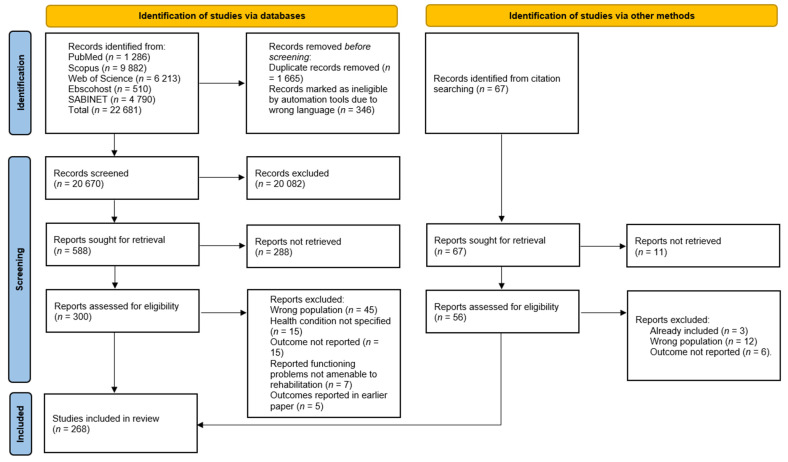
PRISMA flow diagram.

A total of 268 studies met the eligibility criteria. The process of selection of articles at each phase and the reasons for excluding articles are detailed in the PRISMA flow diagram (Figure 1) [40].

### 3.1. Study Characteristics

The sample sizes totalled to 242,085 study participants. The mean age for the included sample could not be calculated as some studies did not report age as a mean (i.e., either reported the median or ranges of age groups). Almost half of the studies (47%) were conducted at primary care level (which included the out-patient clinics of secondary or tertiary hospitals), 22% were conducted in in-patient wards at hospitals, 13% in the community, 9% in specialized hospitals, 7% in rehabilitation facilities while 3% did not report the care level. The geo-location was not reported in 23% of the articles, while 62% of the studies were carried out in urban settings, 11% in rural settings and 3% in peri-urban settings. Most of the studies (43%) were descriptive cross-sectional studies, 28% were cohort studies, 10% were case studies/series, 7% were randomized control trials, while case–control studies, quantitative surveys, quasi-experimental studies, and reviews made up the remaining 12%.

Most studies reported on functioning problems associated with HIV and associated Tuberculosis, stroke, and diabetes mellitus (Table 1).

### 3.2. Prevalence of the Top 20 Functioning Problems

**Figure 2:**
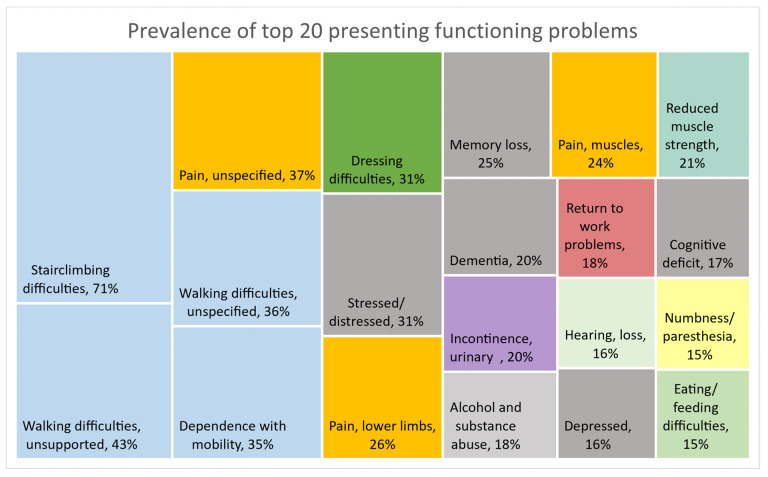
Prevalence (calculated if statistics from at least five articles were available) of top 20 functioning problems from at least 5 articles.

A total of 130 different presenting functioning problems, described by nature and/or body area, were identified from the literature. The reported functioning problems were mostly associated with HIV, Tuberculosis, Diabetes Mellitus and Stroke, and the reported prevalence ranged from 0.1 to 97%. Figure 2 provides an overview of the prevalence of the top 20 functioning problems, considered if at least five articles provided prevalence data, to provide an overview of the most prevalent presenting functioning problems. Mobility problems (shaded blue)—which included difficulty with stairclimbing (70.6%) and difficulties with unsupported walking (43.4%)—and walking difficulties—which were not further described (36.4%)—were most prevalent. Similarly, pain (shaded yellow) of unspecified nature (37.3%), muscle pain (24.3%) and pain that affected the lower limbs (26.4%) had high prevalence. Mental health problems (shaded grey) such as stress, depression, loss of memory and cognitive deficit had prevalence ranging between 15.9% and 30.7%. Respiratory symptoms were not highly prevalent in these populations.

### 3.3. Breakdown of the Most Prevalent Problems

The most prevalent functioning problems (the top 20) mostly fell under mobility, pain, and mental categories of the ICF. To further understand these three most prevalent groups of problems, we report on the other functioning problems that fell under these categories regardless of the number of articles that reported them.

#### 3.3.1. Mobility Problems

**Figure 3:**
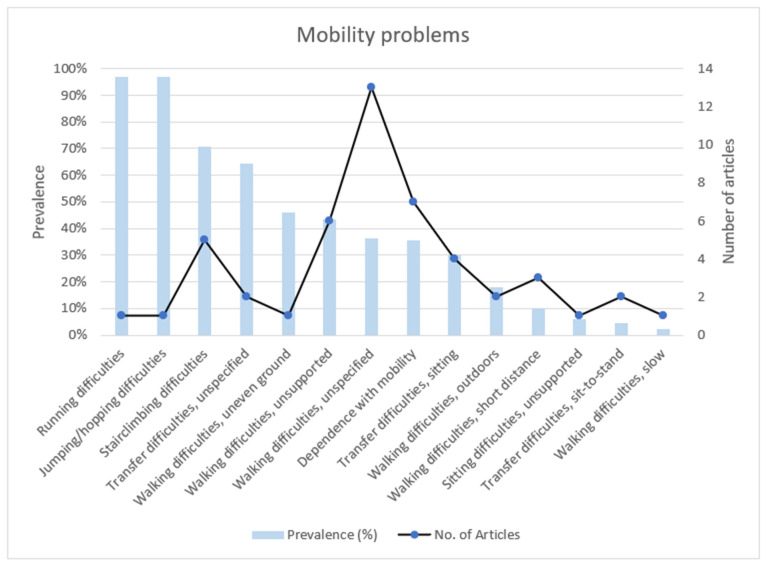
Prevalence and number of articles reporting on mobility problems.

Figure 3 highlights the number of articles and prevalence reported for mobility problems. Articles that reported on mobility problems mostly reported on walking difficulties without giving further description of the problem. The most prevalent mobility problems were difficulties with high-level functioning activities such as jumping/hopping (97%), running (97%) and stairclimbing (70.6%). However, of these high-level functioning activities, only stairclimbing was reported on by at least 5 articles (6 articles). Walking with unsteady gait and standing difficulties were reported by 4 and 3 articles, respectively, but without reporting the prevalence. Related to mobility problems, were falls (7.1% from 7 articles), fear of falling (20%, from 1 article) and balance problems (2.7%, from 4 articles).

#### 3.3.2. Pain Problems

**Figure 4:**
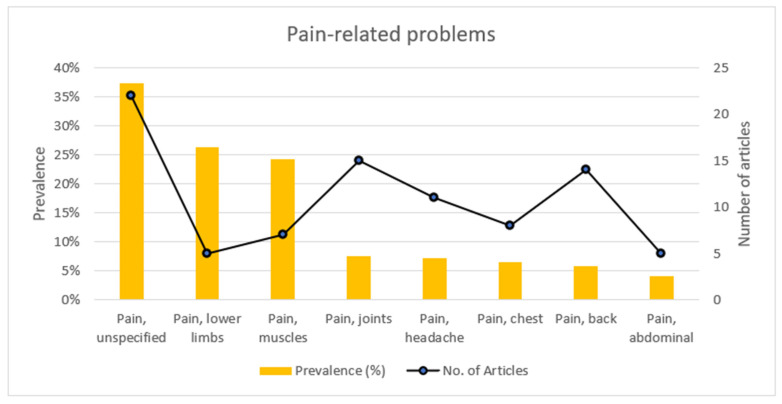
Prevalence of, and number of articles reporting on, pain-related problems.

Figure 4 highlights the number of articles and prevalence reported for pain-related problems. Articles reporting on pain mostly reported on the prevalence of pain but did not specify the nature of the pain or the body area affected. The body area reported to have the highest prevalence of pain (26.4%) were the lower limbs. Pain of muscular origin had a higher prevalence than joint pain (24.3% versus 7.5%). Allodynia and pain of the upper limbs were reported by one article each but without reporting the prevalence.

#### 3.3.3. Mental Health Problems

**Figure 5:**
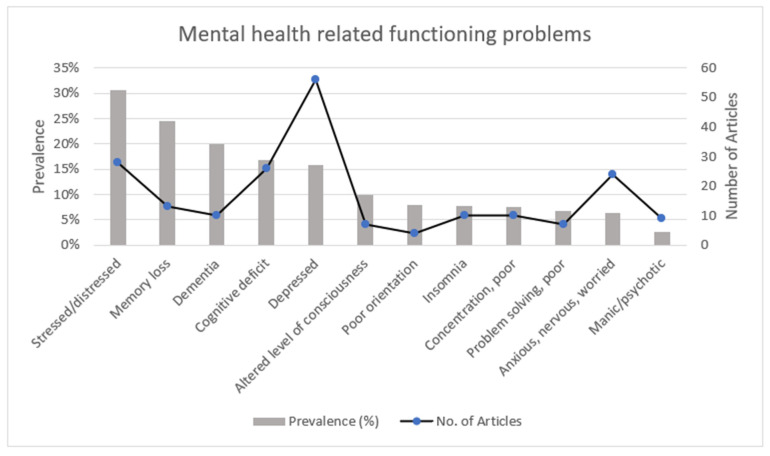
Prevalence of, and number of articles reporting on, mental health related functioning problems.

The most reported mental health problem (shown in Figure 5) was being depressed, reported on by 56 articles. The most prevalent mental health related functioning problem was stress (30.7%). Associated behavioural problems included alcohol and substance abuse, which had a prevalence of 18% and was reported by 31 articles. Suicidal ideation had a low prevalence of 2.3% and was reported on by 13 articles.

### 3.4. Mapping to the ICF

**Figure 6:**
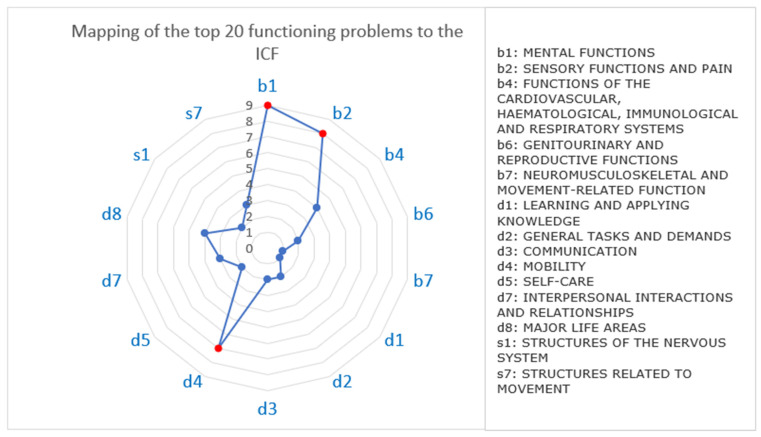
Mapping of the top 20 presenting functioning problems to the ICF domains.

The top 20 presenting functioning problems were mapped to the ICF domains and categories using the outcome measures used in the studies (Table 2). For example, Parker et al. [139] used the Brief Pain Inventory to explore the impact of pain on sleep. The identified functioning problems covered five of the nine body function domains, two of the nine body structure domains and six of the 10 activity limitation and participation domains. Most of the presenting functioning problems affected b1 mental functions (*n* = 9), b2 sensory functions and pain (*n* = 8), and d4 mobility (*n* = 7) (Figure 6). Some functioning problems spanned across several ICF domains—for example, unspecified pain and return to work problems spanned across seven, cognitive deficits and depression spanned across six and five domains, respectively. Most studies provided sufficient information to code the identified functioning problems as far as the fourth level. For example, to add further detail on mobility problems, the walking distance was specified, e.g., d4501 walking long distance, the location was specified, e.g., d4600 moving around within the home. No ICF code was found to indicate walking speed.

## 4. Discussion

This scoping review established the current evidence from peer-reviewed literature on the types and prevalence of functioning problems contributing to most disability in South African adult populations. The most prevalent functioning problems presenting in the South African adult population when mapped to the ICF were mostly mobility-, pain-, and mental health-related. Most studies were found to be at primary care level highlighting the complexities of functioning problems brought about by the associated health conditions experienced by adult patients seen at this level of care.

The prevalence of functioning problems associated with health conditions in the current study was found to be comparable to studies conducted in similar low-resource settings. For example, the current study reported a high prevalence of 70.2% for stairclimbing difficulties compared to Matter et al. [305] who reported 66.7% of adults in Botswana and 65.1% in Swaziland with difficulty with walking or climbing steps. Functional limitations do not always culminate into functioning problems where social support and the required assistive devices are readily available [306]. Thus, it is imperative to scale-up rehabilitation services in low-resource settings to cater for the specific population needs [26].

A total of 130 different functioning problems were identified. The current disease-oriented approach to health care and highly fragmented health care system often leads to ineffective and sub-optimal rehabilitation care [14]. A person-centred approach will effectively address the wide array of functioning problems associated with health conditions [307]. Most functioning problems are inter-related, therefore, by addressing one functioning problem, it is possible to address the other affected domains. For example, addressing balance problems caused by vestibular or muscle weakness may address walking problems that could in turn solve selfcare and participation issues. The identified functioning problems can be used to create awareness among rehabilitation clinicians of the contextual presenting functioning problems to identify, assess, and provide targeted patient-centred treatment plans. An understanding of individual functioning will provide a clearer picture of the burden associated with a health condition and the impact of the health condition on a persons’ life roles or livelihood [308]. Patients too will have better awareness of their own functioning and rehabilitation needs which may have otherwise been dismissed as normal during illness or ageing, empowering them to become informed decision-makers in their healthcare. Thus, rehabilitation clinicians can work with patients to successfully re-integrate patients into their communities [307].

Mapping the identified functioning problems to the ICF highlighted that most functioning problems spanned across several domains and categories of the ICF. For example, pain spanned affected eight different domains of the ICF, which included mobility, interpersonal relationships and major life areas of education and employment. Rehabilitation alone cannot address the full range of educational, social, and development issues [10]. Collaboration with other sectors (such as labour and transport) is required to address the full spectrum of rehabilitation needs. However, rehabilitation remains the key health strategy with the distinctive role of restoring optimal function and alleviating the effects of living with a health condition, in addition to promotive, preventative, curative and palliative roles. South Africa’s NHI drive supports the strengthening of rehabilitation as a health strategy by improving the access and quality of rehabilitation services particularly at primary care [309]. The current study findings are potentially useful in informing the relevant stakeholders and policymakers regarding rehabilitation service planning and strengthening of rehabilitation services.

Rehabilitation service planning: This reasonable approximation of rehabilitation needs within the South African adult population provided by this review may be useful in forecasting the rehabilitation workforce capacity and workforce competencies required in these contexts. Our study findings suggest that at least 7 in 10 adult patients have a functioning problem amenable to rehabilitation—and mostly in patients seeking primary care. This provides a sobering picture of the reality of the complexity and immensity of rehabilitation needs resulting from the high prevalence and wide range of functioning problems in the adult populations presenting to primary care. Yet, only 6–20% of PHC facilities provide rehabilitation services in South Africa [16]. The NHI seeks to provide adequate and relevantly skilled health workers especially at primary care [18]. Thus, the established priority functioning problems associated with health conditions may be useful indicators of rehabilitation need requisite for human resource planning, with respect to both quality and quantity.

The rehabilitation workforce needs to be competent in the knowledge, skills and attitudes required to serve the specific population needs of these low resourced settings. This is especially so for recently qualified community service therapists who are often the sole rehabilitation providers at PHC in South Africa [310]. The WHO’s RCF [311] may be operationalized based on these context-relevant rehabilitation needs. Rehabilitation continuous development and undergraduate rehabilitation curricula may be revised to fill the identified gaps in skills, knowledge and attitudes required to address these key functioning problems presenting in the South African populations. Similarly, clinical practice guidelines which have been reported to be inadequate or contextually irrelevant may be developed [312]. Because CPG need to be brief and succinct to improve uptake among busy clinicians [313], they may be based on these most prevalent functioning problems and continuously revised to adapt to the changing population demographics. This helps to ensure that the scope of such guidelines is suited to the context and needs of South African populations.

Meanwhile, innovative strategies such as task shifting or sharing may be more feasible in low-resource settings which remain chronically plagued by inadequate human resources or additional staff [314,315]. The descriptions of priority functioning problems identified through the current study may provide guidance on the bare minimum skills or knowledge related to mobility, pain and mental health required by each health care provider. The already available primary care workers are favourably positioned to provide the spectrum of care required to meet the increasing and diverse demands of the population’s health needs [316]. Depending on the level of cadre, training may be provided to ensure adequate identification, referral, or basic management (such as patient education).

Strengthening rehabilitation services: The limited resources in LMICs require that financial resource planning be determined by priority needs. The contextual mapping of the most prevalent functioning problems provided in this study will help ensure that patients access the needed rehabilitation services without suffering financial hardship. It may be useful to have allocated funding within the health systems for rehabilitation of functioning problems. For example, in our study, mobility problems were found to be the most prevalent functioning problem. Many factors contribute to mobility problems requiring collaboration between all health care professionals including all disciplines of rehabilitation at primary care [317]. Foremost is the need for assistive devices, which have been reported to be inadequate in most poorly resourced public healthcare facilities in LMICs, one study [318] reporting that 36% of people requiring assistive devices in Zimbabwe acquired them but two thirds of these having to source them privately. Having funding allocated to mobility problems would ensure acquisition of adequate assistive devices and other rehabilitation services. Thus, the findings from this study will prove valuable in building a rehabilitation investment case for funders, healthcare managers and policymakers to make informed decisions with regard to providing both adequate finance and rehabilitation health workforce in support of strengthening rehabilitation at primary care. In the South African context, the ICF framework has been approved for integration into the coding system for the NHI, in addition to the ICD-11 and International Classification of Health Interventions (ICHI). Therefore, the priority functioning problems, mapped to the ICF, and the identified outcome measures will be useful in informing this process.

Mental health-related functioning problems were also found to be, not only highly prevalent in South African adult populations, but also the most reported-on in the reviewed literature. This could have been because mental health is a separate niche of study for specialized professionals including psychologists and psychiatrists. However, there is still lack of prioritisation of resources and policies in response to mental disorders especially at PHC in LMICs including South Africa [319]. The current initiative of clinical guidelines designed to support primary care health workers’ clinical decision-making, namely Primary Care 101, makes little or no reference to rehabilitation in the identification and management of mental health problems [320]. The role of psychosocial rehabilitation is mostly assigned to an auxiliary social worker. However, other health rehabilitation disciplines play significant roles in preventing the onset of mental health problems and facilitating recovery for those experiencing mental health problems, including workplace based mental health programs [321], cognitive rehabilitation interventions, family support and social networks, and physical activity interventions to promote functional independence [322]. Additionally, improved mental health literacy among the patients and caregivers may lead to better treatment planning and health outcomes.

### 4.1. Strengths and Limitations of the Study

The scoping review provided a comprehensive mapping of functioning problems reported in published scientific literature for adult populations in low resource settings. The ICF which is a standard framework for reporting health and functioning states was used. While our study reported comprehensively on our methodology, the repeatability in other countries will be affected by the availability of relevant peer-reviewed publications on functioning problems related to specified health conditions.

The use of the web-based application *Rehab4all* enabled a transparent process of extracting the data used. Additionally, this will allow continued updating of evidence as advances in healthcare or reduced health inequities will influence the profile of priority functioning problems in the populations. For example, hearing loss may no longer be a priority functioning loss as new drug treatments with less ototoxic side-effects, e.g., Bedaquiline versus Kanamycin become more available in low-resource settings [197]. Other countries may access or contribute to the data to provide better comparisons of contextually similar settings.

The calculated prevalence was dependent on the number of articles provided. Not all functioning problems were reported on by several articles thus affecting the validity of our results. A statistician or data scientist can be engaged to employ more advanced techniques to analyse the burden of the identified functioning problems and control for confounding factors such as publication bias.

The high proportion of HIV-related functioning problems in effect reflects how the sub-Saharan region continues to be the greatest contributor to the HIV epidemic despite decreased incidence, therefore, there continues to be global research interest and funding opportunities towards HIV-related research. South Africa’s public sector health research funding remains below the proposed 2% of the national health budget [323]. However, more research is needed in other health conditions.

### 4.2. Further Research

Additional research such as a qualitative study may be important to validate the study findings and fill the gaps in findings especially regarding participation restrictions. Cross-sectional studies may be done to determine the prevalence of the functioning problems that were reported qualitatively such as in case studies or case series.

Environmental factors were not considered in this review as this requires a more extensive review of the barriers and facilitators of functioning within the selected contexts.

The prevalence of multimorbidity is high in South Africa [15]. The review could be taken further into understanding the functioning problems associated with common multimorbidity patterns. Authors should discuss the results and how they can be interpreted from the perspective of previous studies and of the working hypotheses. The findings and their implications should be discussed in the broadest context possible. Future research directions may also be highlighted.

Considering the enormity of the review, we pragmatically selected the top ten health conditions contributing to greatest disease burden in South Africa based on the GBD 2019 estimates [8]. Thus, we may have missed notable health conditions in South Africa such as neurodegenerative disorders that are especially associated with HIV and ageing. Except for dementia, the lack of standardized and widely accepted screening and diagnostic tools/criteria which facilitate epidemiological studies of other common neurodegenerative conditions, e.g., Guillain Barre Syndrome or Parkinsonism may be reason for the lack of evidence regarding their prevalence in South Africa [324]. Similarly, our review was limited to adult populations. As a result, genetic diseases which are more highly prevalent in children and young adults were not included. Perhaps future reviews may address these health conditions and/or populations.

## 5. Conclusions

The study has provided an example of how other poorly resourced countries can leverage on already published evidence on functioning problems as a first step in a transparent process towards informed planning in strengthening rehabilitation within health systems. The scoping review has identified the most prevalent functioning problems associated with health conditions contributing to most disability in South Africa. The wide range of highly prevalent functioning problems were mostly reported in populations at primary care. The most common problems were associated with mobility, mental health, and pain. This points towards targeted planning of innovative strategies towards strengthening rehabilitation service delivery at primary care to address these complexities where there is an inadequate rehabilitation workforce.

Early detection of these key functioning problems by the health system at primary care can be facilitated by routine screening for the identified functioning problems through available and contextually appropriate easy to use tools. The country’s PHC system can be re-engineered to provide health promotion and prevention services that target the key functioning problems. This can be achieved through effective knowledge dissemination and self-management strategies within the communities as well as training for the available primary care cadre to provide brief interventions or appropriate referral for rehabilitation care. These moderate changes to will ultimately result in improved population health outcomes and achieving universal health coverage for disadvantaged populations in poorly resourced settings.

## Figures and Tables

**Table 1:** Number of articles according to the top ten health conditions contributing to greatest disability in South Africa.

Condition	Sub-Category	No of Articles (*n*)	Subtotals
HIV	HIV only [41,42,43,44,45,46,47,48,49,50,51,52,53,54,55,56,57,58,59,60,61,62,63,64,65,66,67,68,69,70,71,72,73,74,75,76,77,78,79,80,81,82,83,84,85,86,87,88,89,90,91,92,93,94,95,96,97,98,99,100,101,102,103,104,105,106,107,108,109,110,111,112,113,114,115,116,117,118,119,120,121,122,123,124,125,126,127,128,129,130,131,132,133,134,135,136,137,138,139,140,141,142,143,144,145,146,147,148,149,150,151,152,153,154,155,156,157,158,159,160,161,162,163]	126	
	HIV with TB [164,165,166,167,168,169,170,171,172,173]	10	
	HIV with Kaposi’s sarcoma [174,175]	2	
	HIV with Cryptococcal Meningocele [176]	1	139
Tuberculosis	TB, unspecified [148,177,178,179,180,181,182,183,184,185,186,187,188,189,190]	15	
	TB, MDR [191,192,193,194,195,196,197,198,199,200]	10	
	TB, pulmonary [201,202,203]	3	
	TB, spinal [204,205,206]	3	
	TB, tuberculoma [207]	1	
	TB, meningitis [208]	1	33
Stroke	Stroke, unspecified [148,209,210,211,212,213,214,215,216,217,218,219,220,221,222,223,224,225,226,227,228,229,230,231]	24	24
Diabetes Mellitus	DM, Type unspecified [232,233,234,235,236,237,238,239]	8	
	DM, Type II [240,241,242,243,244,245,246]	7	
	DM, Type I [241,242]	2	
	DM with hypertension [247]	1	
	DM with HIV [248]	1	19
Spinal pain	Neck pain [249,250,251,252,253,254,255,256]	8	
	LBP, mechanical [252,254,257,258,259,260]	6	
	LBP, pathological [261,262,263]	3	17
Fracture/dislocation	Lower limb fracture/dislocation [264,265,266,267,268]	5	
	Upper limb fracture/dislocation [269,270,271,272,273]	5	
	Pathological fracture(s) [274,275]	2	
	Spinal fracture(s) [276,277]	2	
	Rib fracture(s) [278]	1	
	Skull/cranial fracture(s) [279]	1	
	Fracture, unspecified [280]	1	17
Arthritis	Rheumatoid arthritis [281,282,283,284,285]	5	
	OA, hip [286]	1	
	OA, knee [287]	1	
	OA, other [237]	1	8
Burns	Burns only [288,289,290,291,292]	5	
	Burns with amputation [293]	1	6
Hearing loss	Hearing loss [293,294,295]	3	3
Headaches	Headache, cervicogenic [296]	1	
	Headache, unspecified [254]	1	2

DM—diabetes mellitus, HIV—human immunodeficiency virus, LBP—low back pain, OA—osteoarthritis, TB—tuberculosis, TB, MDR—multidrug resistant tuberculosis. Background color: Pathological-blue, Arthritis—blue; Hearing loss -blue, Headache—unshaded and LBP-shaded.

**Table 2:** Mapping of top 20 presenting functioning problems to the ICF according to related outcome measures.

Problem	ICF Domain	ICF Code/Category	Outcome Measures
Stairclimbing difficulties	d4 MOBILITY	d455 moving around	Barthel Index (BI); [211,216,217] Rivermead Motor Assessment scores of Gross function (RMA-G); Nottingham extended activities of daily living (NEADL) scale [297]
Walking difficulties, unsupported	d4 MOBILITY	d450 Walking d465 Moving around using equipment	Rivermead Mobility Index (RMI); [215] Berg Balance Scale (BBS); [215] Postural Assessment Scale for Stroke Patients (PASS); [215] self-report; [76] Use of assistive devices [227]
Pain, unspecified	b1 MENTAL FUNCTIONS	b134 Sleep functions b152 Emotional functions	Wisconsin Brief Pain Questionnaire (WBPQ); [125] Beck Depression Inventory (BDI); [139] Pain Catastrophizing Scale (PCS); [150,249] Hopkins Symptoms Checklist-25 (HSCL-25); [150] Neuropathic Diagnostic Questionnaire (DN4) [249]
	b2 SENSORY FUNCTIONS AND PAIN	b270 Sensory functions related to temperature and other stimuli b280 Sensation of pain	Brief Pain Inventory (Xhosa) (BPI-Xhosa); [298] WBPQ; [125,150] Brief Neuropathy Screening Tool (BNST); [150,299] Visual Analogue Scale (VAS), [280] Numeric Pain Rating Scale (NPRS); [249] Brief Peripheral Neuropathy Screen (BPNS) [75]
	s7 STRUCTURES RELATED TO MOVEMENT	s750 Structure of lower extremity	ASAMI score, the Foot Function Index (FFI), radiographic outcomes; [300] The Lower Extremity Functional Scale (LEFS) [75]
	d2 GENERAL TASKS AND DEMANDS	d240 Handling stress and other psychological demands	HSCL-25 [150]
	d4 MOBILITY	d450 Walking	(BPI [130]; Karnofsky Performance Scale (KPS) [125]
	d7 INTERPERSONAL INTERACTIONS AND RELATIONSHIPS	d710 Basic interpersonal interactions	BPI [130]
	d8 MAJOR LIFE AREAS	d820 School education d845 Acquiring, keeping, and terminating a job	BPI [130]
Walking difficulties, unspecified	b2 SENSORY FUNCTIONS AND PAIN	b235 Vestibular functions	Self-report [76]
	d4 MOBILITY	d450 Walking	Self-report [76]
Dependence with mobility	d4 MOBILITY	d450 Walking d465 Moving around using equipment	Rankin scores; [301] BI; [211] Timed-up-and-go-test; [182] BBS; [215] Nurick classification [205]
Dressing difficulties	d5 SELF-CARE	d540 Dressing	BI; [211] World Health Organization’s Disability Assessment Schedule 2.0 (WHODAS 2.0) [209]
Stressed/distressed	b1 MENTAL FUNCTIONS	b126 Temperament and personality functions	Questionnaire on Stress in Diabetes—Revised (QSD-R); [244] Breslau PTSD screener [61]
	d2 GENERAL TASKS AND DEMANDS	d240 Handling stress and other psychological demand	Primary Care PTSD Screen (PC-PTSD); [179] The Kessler Psychological Distress Scale (K-10); [179] Mini-International Neuropsychiatric Interview (MINI); [135] Hopkins Symptom Checklist (HSCL); [101] Composite International Diagnostic Interview (CIDI); [78] CIDI [111]
	d7 INTERPERSONAL INTERACTIONS AND RELATIONSHIPS	d750 Informal social relationships	Questionnaire on Stress in Diabetes—Revised (QSD-R) [244]
Pain, lower limbs	b2 SENSORY FUNCTIONS AND PAIN	b280 Sensation of pain	BPI-Xhosa; [138] NPRS; [291] BPNS; [64] Visual Analogue Scale (VAS) [280]
	s7 STRUCTURES RELATED TO MOVEMENT	s750 Structure of lower extremity	BPI-Xhosa; [57,138] Burn Specific Pain and Anxiety scale (BSPAS) [291]
Memory loss	b1 MENTAL FUNCTIONS	b117 Intellectual functions b144 Memory functions b164 Higher-level cognitive functions	WHODAS 2.0; [209] International HIV Dementia Scale (IHDS); [93] Neuropsychological (NP) test battery; [132] The revised signs and symptoms checklist for persons with HIV disease (SSC-HIVrev); [146] The International Classification of Functioning, Disability and Health (ICF) [128]
Pain, muscles	b2 SENSORY FUNCTIONS AND PAIN	b280 Sensation of pain	ICF; [128] WHODAS 2.0; [107] self-report; [265] SSC-HIVrev [79]
Reduced muscle strength	b4 FUNCTIONS OF THE CARDIOVASCULAR, HAEMATOLOGICAL, IMMUNOLOGICAL AND RESPIRATORY SYSTEMS	b455 Exercise tolerance functions	ICF; [128]
	b7 NEUROMUSCULOSKELETAL AND MOVEMENT-RELATED FUNCTION	b710 Mobility of joint functions b730 Muscle power functions	ICF; [128] Oxford Scale of muscle strength; [42] isokinetic dynamometry; [269,283] sphygmomanometer [283]
	s7 STRUCTURES RELATED TO MOVEMENT	s750 Structure of lower extremity s720 Structure of shoulder region	ICF; [128] isokinetic dynamometry [269,283]
Dementia	b1 MENTAL FUNCTIONS	b117 Intellectual functions b144 Memory functions b147 Psychomotor functions b152 Emotional functions	NP test battery; [132] IHDS; [80,155,200] mini-mental state examination (MMSE), Montreal cognitive assessment (MOCA), Simioni symptom questionnaire (SSQ) and cognitive assessment tool-rapid version (CAT-rapid) [95]
Incontinence, urinary	b6 GENITOURINARY AND REPRODUCTIVE FUNCTIONS	b620 Urination functions	Self-report [198] BI; [211,217] Survey of Autonomic Symptoms (SAS) [75]
Alcohol and substance abuse	b1 MENTAL FUNCTIONS	b117 Intellectual functions b130 Energy and drive functions b152 Emotional functions	MINI; [155] Alcohol Use Disorder Identification Test (AUDIT); [91,147,189] CAGE questionnaire; [54] Alcohol, Smoking and Substance Involvement Screening Test questionnaire (ASSIST) [102]
Return to work problems	b1 MENTAL FUNCTIONS	b117 Intellectual functions b130 Energy and drive functions b144 Memory functions b152 Emotional functions b167 Mental functions of language	Return to work questionnaire [212,226]
	b2 SENSORY FUNCTIONS AND PAIN	b210 Seeing functions b280 Sensation of pain	Return to work questionnaire; [212,226] BPI [212,226]
	b4 FUNCTIONS OF THE CARDIOVASCULAR, HAEMATOLOGICAL, IMMUNOLOGICAL AND RESPIRATORY SYSTEMS	b455 Exercise tolerance functions	Return to work questionnaire [212,226]
	b6 GENITOURINARY AND REPRODUCTIVE FUNCTIONS	b620 Urination functions	Return to work questionnaire [212,226]
	d3 COMMUNICATION	d330 Speaking	Return to work questionnaire [212,226]
	d4 MOBILITY	d445 Hand and arm use d450 Walking d470 Using transportation	Return to work questionnaire [212,226]
	d8 MAJOR LIFE AREAS	d845 Acquiring, keeping, and terminating a job	Return to work questionnaire [212,226,250]
Cognitive deficit	b1 MENTAL FUNCTIONS	b117 Intellectual functions b140 Attention functions b144 Memory functions b147 Psychomotor functions b156 Perceptual functions b160 Thought functions b164 Higher-level cognitive functions	NP test battery; [157] MoCA; [156] Tampa Scale for Kinesiophobia-11 (TSK-11); PCS; [250] NeuroScreen Performance [157]; IHDS; [80,155,200] MMSE, SSQ and cognitive assessment tool-rapid version (CAT-rapid); [95] Bedside Executive Screening Test (BEST) [245]
	b3 VOICE AND SPEECH FUNCTIONS	b330 Fluency and rhythm of speech functions	NP test battery; [45,157,171]
	b4 FUNCTIONS OF THE CARDIOVASCULAR, HAEMATOLOGICAL, IMMUNOLOGICAL AND RESPIRATORY SYSTEMS	b435 Immunological system functions	Laboratory assessments for immune markers TYMP and NGAL [58]
	d4 MOBILITY	d475 Driving	NP test battery and driving simulations [82]
	d8 MAJOR LIFE AREAS	d820 School education	NP test battery; [157]
	s1 STRUCTURES OF THE NERVOUS SYSTEM	s110 Structure of brain	Brain imaging [302]
Hearing, loss	b2 SENSORY FUNCTIONS AND PAIN	b167 Mental functions of language b230 Hearing functions	Otoscopy, tympanometry, pure-tone audiometry, and distortion product otoacoustic emissions [66,105,184,191,197]
	d1 LEARNING AND APPLYING KNOWLEDGE	d166 Reading	South African Sign Language (SASL) [303]
	d3 COMMUNICATION	d330 Speaking	SASL [303]
Depressed	b1 MENTAL FUNCTIONS	b126 Temperament and personality functions b130 Energy and drive functions b134 Sleep functions b147 Psychomotor functions b152 Emotional functions	NP test battery; Patient Health Questionnaire (PHQ-9); [154] Centers for Epidemiologic Studies Depression Scale (CES-D) [108,144] Hospital Anxiety and Depression Scale (HADS) [243] Beck Depression Inventory—Second Edition (BDI II) [133]
	b2 SENSORY FUNCTIONS AND PAIN	b280 Sensation of pain	PHQ-9; [154]
	b4 FUNCTIONS OF THE CARDIOVASCULAR, HAEMATOLOGICAL	b455 Exercise tolerance functions	PHQ-9; [154]
	d7 INTERPERSONAL INTERACTIONS AND RELATIONSHIPS	d710 Basic interpersonal interactions d770 Intimate relationships	Edinburgh Postnatal Depression Scale (EPDS) [60,131]
	d8 MAJOR LIFE AREAS	d845 Acquiring, keeping, and terminating a job d850 Remunerative employment	BPI [57]
Numbness/paraesthesia	b2 SENSORY FUNCTIONS AND PAIN	b265 Touch function b270 Sensory functions related to temperature and stimuli b280 Sensation of pain b840 Sensation related to the skin	Total Neuropathy Score (TNSr) [64] SCC- HIVrev; [304] Assessment of fine touch, pin-prick and vibration sense for neuropathy symptom score (NSS) and neuropathy disability score (NDS); [192] AIDS Clinical Trials Group BPNS [56]
	s1 STRUCTURES OF THE NERVOUS SYSTEM	s120 Spinal cord and related structures	Radiological imaging
Eating/feeding difficulties	d5 SELF-CARE	d550 Eating	National Institute of Health Stroke Scale, (NIHSS); [217] WHODAS 2.0; [209] South African dysphagia screening tool (SADS); [213] Self-report [215]

## Data Availability

The data presented in this study are available on request from the corresponding author. The data are not publicly available due to privacy restrictions of *Rehab4all* application developed.

## Supplementary Materials

The following supporting information can be downloaded at: https://www.mdpi.com/article/10.3390/ijerph192315636/s1. File S1: Preferred Reporting Items for Systematic reviews and Meta-Analyses extension for Scoping Reviews (PRISMA-ScR) Checklist; File S2: Full electronic search strategy for all databases.
