# Supplementary material for: Functioning Problems Associated with Health Conditions with Greatest Disease Burden in South Africa: A Scoping Review
Source: Int J Environ Res Public Health. 2022 Nov 24;19(23):15636. doi: 10.3390/ijerph192315636 (PMC9735592; doi:10.3390/ijerph192315636)
Supplement: Supplementary file 1 [file ijerph-19-15636-s001.zip › S2_Full search strategies_IJERPH.pdf]

Supplementary file 1: Search strategies (and hits) per database

Pubmed (06/08/2021); updated (14/06/2022)

(((stroke[MeSH Terms]) OR (Stroke*[Title/Abstract] OR cerebrovascular accident*[Title/Abstract] OR CVA[Title/Abstract] OR brain vascular accident*[Title/Abstract] OR apoplexy[Title/Abstract] OR brain infarct*[Title/Abstract] OR ((brain[Title/Abstract] OR cerebr*[Title/Abstract] OR cerebell*[Title/Abstract] OR intracran*[Title/Abstract] OR intracerebral[Title/Abstract] OR subarachnoid) NEAR/3 (ischemi*[Title/Abstract] OR infarct*[Title/Abstract] OR thrombo*[Title/Abstract] OR emboli*[Title/Abstract] OR occlus*[Title/Abstract] OR hypoci*[Title/Abstract] OR h?emorrhage*[Title/Abstract])))) AND (Impairment*[Title/Abstract] OR "functional loss*"[Title/Abstract] OR weak*[Title/Abstract] OR defic*[Title/Abstract] OR disab*[Title/Abstract] OR disorder*[Title/Abstract] OR dysfunction[Title/Abstract] OR function*[Title/Abstract] OR frail*[Title/Abstract] OR "function* limitation*"[Title/Abstract] OR restriction*[Title/Abstract] OR handicap*[Title/Abstract] OR "activity limitation*"[Title/Abstract] OR "participation restriction*"[Title/Abstract])) AND ("South Africa*"[Text Word]) Filters: Humans, Afrikaans, English, Adult: 19+ years, from 2006 – 2021 **71**

(((HIV[Title/Abstract] OR HIV-1[Title/Abstract] OR HIV-2[Title/Abstract] OR HIV1[Title/Abstract] OR HIV2[Title/Abstract] OR ("human immun*"[Title/Abstract] AND virus[Title/Abstract]) OR HIV-infected[Title/Abstract] OR "HIV infect*"[Title/Abstract] OR ("acquired immun*"[Title/Abstract] AND syndrom*[Title/Abstract]) OR HIV/AIDS[Title/Abstract]) OR (HIV[MeSH Terms])) AND (Impairment*[Title/Abstract] OR "functional loss*"[Title/Abstract] OR weak*[Title/Abstract] OR defic*[Title/Abstract] OR disab*[Title/Abstract] OR disorder*[Title/Abstract] OR dysfunction[Title/Abstract] OR function*[Title/Abstract] OR frail*[Title/Abstract] OR "function* limitation*"[Title/Abstract] OR restriction*[Title/Abstract] OR handicap*[Title/Abstract] OR "activity limitation*"[Title/Abstract] OR "participation restriction*"[Title/Abstract])) AND ("South Africa*"[Text Word]) Filters: Humans, Afrikaans, English, Adult: 19+ years, from 2006 – 2021 **691**

(((Tuberculosis[MeSH Terms]) OR (Tubercul*[Title/Abstract] OR TB[Title/Abstract])) AND (Impairment*[Title/Abstract] OR "functional loss*"[Title/Abstract] OR weak*[Title/Abstract] OR defic*[Title/Abstract] OR disab*[Title/Abstract] OR disorder*[Title/Abstract] OR dysfunction[Title/Abstract] OR function*[Title/Abstract] OR frail*[Title/Abstract] OR "function* limitation*"[Title/Abstract] OR restriction*[Title/Abstract] OR handicap*[Title/Abstract] OR "activity limitation*"[Title/Abstract] OR "participation restriction*"[Title/Abstract])) AND ("South Africa*"[Text Word]) Filters: Humans, Afrikaans, English, Adult: 19+ years, from 2006 – 2021 **206**

(((Diabetes[MeSH Terms]) OR (Diabet*[Title/Abstract] OR diabetes mellitus[Title/Abstract] OR DM[Title/Abstract] OR IDDM[Title/Abstract] OR NIDDM[Title/Abstract] OR T1DM[Title/Abstract] OR T2DM[Title/Abstract] OR insulin?depend*[Title/Abstract] OR non insulin?depend*[Title/Abstract] OR glucose intoleran*[Title/Abstract] OR insulin resistan*[Title/Abstract])) AND (Impairment*[Title/Abstract] OR "functional loss*"[Title/Abstract] OR weak*[Title/Abstract] OR defic*[Title/Abstract] OR disab*[Title/Abstract] OR disorder*[Title/Abstract] OR dysfunction[Title/Abstract] OR function*[Title/Abstract] OR frail*[Title/Abstract] OR "function* limitation*"[Title/Abstract] OR restriction*[Title/Abstract] OR handicap*[Title/Abstract] OR "activity limitation*"[Title/Abstract] OR "participation restriction*"[Title/Abstract])) AND ("South Africa*"[Text Word]) Filters: Humans, Afrikaans, English, Adult: 19+ years, from 2006 – 2021 **160**

(((Burn[MeSH Terms]) OR (Burn*[Title/Abstract])) AND (Impairment*[Title/Abstract] OR "functional loss*"[Title/Abstract] OR weak*[Title/Abstract] OR defic*[Title/Abstract] OR disab*[Title/Abstract] OR disorder*[Title/Abstract] OR dysfunction[Title/Abstract] OR function*[Title/Abstract] OR frail*[Title/Abstract] OR "function* limitation*"[Title/Abstract] OR restriction*[Title/Abstract] OR handicap*[Title/Abstract] OR "activity limitation*"[Title/Abstract] OR "participation restriction*"[Title/Abstract])) AND ("South Africa*"[Text Word]) Filters: Humans, Afrikaans, English, Adult: 19+ years, from 2006 - 2021 **28**

((("Deafness"[Mesh] OR "Persons With Hearing Impairments"[Mesh]) OR (Hearing[Title/Abstract] OR deaf*[Title/Abstract] OR ototoxic*[Title/Abstract] OR ear[Title/Abstract] OR presbycusis[Title/Abstract])) AND (Impairment*[Title/Abstract] OR "functional loss*"[Title/Abstract] OR weak*[Title/Abstract] OR defic*[Title/Abstract] OR disab*[Title/Abstract] OR disorder*[Title/Abstract] OR dysfunction[Title/Abstract] OR function*[Title/Abstract] OR frail*[Title/Abstract] OR "function* limitation*"[Title/Abstract] OR restriction*[Title/Abstract] OR handicap*[Title/Abstract] OR "activity limitation*"[Title/Abstract] OR "participation restriction*"[Title/Abstract])) AND ("South Africa*"[Text Word]) Filters: Humans, Afrikaans, English, Adult: 19+ years, from 2006 – 2021 **61**

(((Fractures, Bone[MeSH Terms] OR Joint dislocations[MeSH Terms]) OR (Fracture*[Title/Abstract] OR disloc*[Title/Abstract] OR sublux*[Title/Abstract])) AND (Impairment*[Title/Abstract] OR "functional loss*"[Title/Abstract] OR weak*[Title/Abstract] OR defic*[Title/Abstract] OR disab*[Title/Abstract] OR disorder*[Title/Abstract] OR dysfunction[Title/Abstract] OR function*[Title/Abstract] OR frail*[Title/Abstract] OR "function* limitation*"[Title/Abstract] OR restriction*[Title/Abstract] OR handicap*[Title/Abstract] OR "activity limitation*"[Title/Abstract] OR "participation restriction*"[Title/Abstract])) AND ("South Africa*"[Text Word]) Filters: Humans, Afrikaans, English, Adult: 19+ years, from 2006 – 2021 **10**

(((Arthritis, Rheumatoid[MeSH Terms]) OR ("reumatoid arthritis"[Title/Abstract] OR "rheumatoid arthritis"[Title/Abstract] OR "rheumatic arthritis"[Title/Abstract] OR "rheumarthritis"[Title/Abstract])) AND (Impairment*[Title/Abstract] OR "functional loss*"[Title/Abstract] OR weak*[Title/Abstract] OR defic*[Title/Abstract] OR disab*[Title/Abstract] OR disorder*[Title/Abstract] OR dysfunction[Title/Abstract] OR function*[Title/Abstract] OR frail*[Title/Abstract] OR "function* limitation*"[Title/Abstract] OR restriction*[Title/Abstract] OR handicap*[Title/Abstract] OR "activity limitation*"[Title/Abstract] OR "participation restriction*"[Title/Abstract])) AND ("South Africa*"[Text Word]) Filters: Humans, Afrikaans, English, Adult: 19+ years, from 2006 – 2021 **15**

(((Gout[MeSH Terms]) OR (Gout*[Title/Abstract])) AND (Impairment*[Title/Abstract] OR "functional loss*"[Title/Abstract] OR weak*[Title/Abstract] OR defic*[Title/Abstract] OR disab*[Title/Abstract] OR disorder*[Title/Abstract] OR dysfunction[Title/Abstract] OR function*[Title/Abstract] OR frail*[Title/Abstract] OR "function* limitation*"[Title/Abstract] OR restriction*[Title/Abstract] OR handicap*[Title/Abstract] OR "activity limitation*"[Title/Abstract] OR "participation restriction*"[Title/Abstract])) AND ("South Africa*"[Text Word]) Filters: Humans, Afrikaans, English, Adult: 19+ years, from 2006 – 2021 **1**

(((Osteoarthritis, Hip[MeSH Terms] OR Osteoarthritis, Knee[MeSH Terms]) OR (Osteoarthriti*[Title/Abstract] OR osteoarthro*[Title/Abstract] OR gonarthriti*[Title/Abstract] OR gonarthro*[Title/Abstract] OR coxarthriti*[Title/Abstract] OR coxarthro*[Title/Abstract] OR arthros*[Title/Abstract] OR arthrot*[Title/Abstract])) AND (Impairment*[Title/Abstract] OR "functional loss*"[Title/Abstract] OR weak*[Title/Abstract] OR defic*[Title/Abstract] OR disab*[Title/Abstract] OR disorder*[Title/Abstract] OR dysfunction[Title/Abstract] OR function*[Title/Abstract] OR frail*[Title/Abstract] OR "function* limitation*"[Title/Abstract] OR restriction*[Title/Abstract] OR handicap*[Title/Abstract] OR "activity limitation*"[Title/Abstract] OR "participation restriction*"[Title/Abstract])) AND ("South Africa*"[Text Word]) Filters: Humans, Afrikaans, English, Adult: 19+ years, from 2006 – 2021 **7**

(((Neck Pain[MeSH Terms]) OR ("neck pain*"[Title/Abstract] OR "neck ache*"[Title/Abstract] OR neckache*[Title/Abstract] OR "cervical pain*"[Title/Abstract] OR cervicodynia*[Title/Abstract] OR cervicalgia*[Title/Abstract] OR cervicogenic[Title/Abstract] OR whiplash[Title/Abstract] OR torticollis[Title/Abstract])) AND (Impairment*[Title/Abstract] OR "functional loss*"[Title/Abstract] OR weak*[Title/Abstract] OR defic*[Title/Abstract] OR disab*[Title/Abstract] OR disorder*[Title/Abstract] OR dysfunction[Title/Abstract] OR function*[Title/Abstract] OR frail*[Title/Abstract] OR "function* limitation*"[Title/Abstract] OR restriction*[Title/Abstract] OR handicap*[Title/Abstract] OR "activity limitation*"[Title/Abstract] OR "participation restriction*"[Title/Abstract])) AND ("South Africa*"[Text Word]) Filters: Humans, Afrikaans, English, Adult: 19+ years, from 2006 – 2021 **2**

((((Low Back Pain[MeSH Terms]) OR ("Low back pain"[Title/Abstract] OR backache[Title/Abstract] OR "spinal pain"[Title/Abstract] OR lumbago[Title/Abstract] OR coccydynia[Title/Abstract] OR spondylosis[Title/Abstract] OR spondylolisthesis[Title/Abstract] OR stenosis[Title/Abstract] OR disc hernia*[Title/Abstract] OR disc prolapse*[Title/Abstract] OR disc degeneration[Title/Abstract] OR slipped disc[Title/Abstract] OR lumb* pain[Title/Abstract] OR scoliosis[Title/Abstract])) AND (Impairment*[Title/Abstract] OR "functional loss*"[Title/Abstract] OR weak*[Title/Abstract] OR defic*[Title/Abstract] OR disab*[Title/Abstract] OR disorder*[Title/Abstract] OR dysfunction[Title/Abstract] OR function*[Title/Abstract] OR frail*[Title/Abstract] OR "function* limitation*"[Title/Abstract] OR restriction*[Title/Abstract] OR handicap*[Title/Abstract] OR "activity limitation*"[Title/Abstract] OR "participation restriction*"[Title/Abstract]))) AND ("South Africa*"[Text Word]) Filters: Humans, Afrikaans, English, Adult: 19+ years, from 2006 – 2021 **18**

(((Headache Disorders, Primary[MeSH Terms]) OR (Headache*[Title/Abstract] OR migraine*[Title/Abstract] OR cephalgia*[Title/Abstract] OR cephalalgi*[Title/Abstract])) AND (Impairment*[Title/Abstract] OR "functional loss*"[Title/Abstract] OR weak*[Title/Abstract] OR defic*[Title/Abstract] OR disab*[Title/Abstract] OR disorder*[Title/Abstract] OR dysfunction[Title/Abstract] OR function*[Title/Abstract] OR frail*[Title/Abstract] OR "function* limitation*"[Title/Abstract] OR restriction*[Title/Abstract] OR handicap*[Title/Abstract] OR "activity limitation*"[Title/Abstract] OR "participation restriction*"[Title/Abstract])) AND ("South Africa*"[Text Word]) Filters: Humans, Afrikaans, English, Adult: 19+ years, from 2006 – 2021 **16**

Total hits for PubMed **1286**

Scopus (11/08/2021); updated (17/06/2022)

TITLE-ABS-KEY(Stroke* OR "cerebrovascular accident*" OR CVA) AND TITLE-ABS-KEY(Impairment* OR "functional loss*" OR weak* OR defic* OR disab* OR disorder* OR dysfunction OR function* OR frail* OR "function* limitation*" OR restriction* OR handicap* OR "activity limitation*" OR "participation restriction*") AND ALL("South Africa*") AND (LIMIT-TO (AFFILCOUNTRY,"South Africa") OR LIMIT-TO (AFFILCOUNTRY,"Undefined")) AND (LIMIT-TO (PUBYEAR,2021) OR LIMIT-TO (PUBYEAR,2020) OR LIMIT-TO (PUBYEAR,2019) OR LIMIT-TO (PUBYEAR,2018) OR LIMIT-TO (PUBYEAR,2017) OR LIMIT-TO (PUBYEAR,2016) OR LIMIT-TO (PUBYEAR,2015) OR LIMIT-TO (PUBYEAR,2014) OR LIMIT-TO (PUBYEAR,2013) OR LIMIT-TO (PUBYEAR,2012) OR LIMIT-TO (PUBYEAR,2011) OR LIMIT-TO (PUBYEAR,2010) OR LIMIT-TO (PUBYEAR,2009) OR LIMIT-TO (PUBYEAR,2008) OR LIMIT-TO (PUBYEAR,2007) OR LIMIT-TO (PUBYEAR,2006)) AND (LIMIT-TO (LANGUAGE,"English")) **757**

TITLE-ABS-KEY(HIV OR HIV-1 OR HIV-2 OR HIV1 OR HIV2 OR ("human immun*" AND virus) OR HIV-infected OR "HIV infect*" OR ("acquired immun*" AND syndrom*) OR HIV/AIDS) AND TITLE-ABS-KEY(Impairment* OR "functional loss*" OR weak* OR defic* OR disab* OR disorder* OR dysfunction OR function* OR frail* OR "function* limitation*" OR restriction* OR handicap* OR "activity limitation*" OR "participation restriction*") AND ALL("South Africa*") AND (LIMIT-TO (AFFILCOUNTRY,"South Africa")) AND (LIMIT-TO (PUBYEAR,2021) OR LIMIT-TO (PUBYEAR,2020) OR LIMIT-TO (PUBYEAR,2019) OR LIMIT-TO (PUBYEAR,2018) OR LIMIT-TO (PUBYEAR,2017) OR LIMIT-TO (PUBYEAR,2016) OR LIMIT-TO (PUBYEAR,2015) OR LIMIT-TO (PUBYEAR,2014) OR LIMIT-TO (PUBYEAR,2013) OR LIMIT-TO (PUBYEAR,2012) OR LIMIT-TO (PUBYEAR,2011) OR LIMIT-TO (PUBYEAR,2010) OR LIMIT-TO (PUBYEAR,2009) OR LIMIT-TO (PUBYEAR,2008) OR LIMIT-TO (PUBYEAR,2007) OR LIMIT-TO (PUBYEAR,2006)) AND (LIMIT-TO (LANGUAGE,"English") OR LIMIT-TO (LANGUAGE,"Afrikaans")) **2681**

TITLE-ABS-KEY(tubercul* OR TB) AND TITLE-ABS-KEY(Impairment* OR "functional loss*" OR weak* OR defic* OR disab* OR disorder* OR dysfunction OR function* OR frail* OR "function* limitation*" OR restriction* OR handicap* OR "activity limitation*" OR "participation restriction*") AND ALL("South Africa*") AND (LIMIT-TO (AFFILCOUNTRY,"South Africa")) AND (LIMIT-TO (PUBYEAR,2021) OR

LIMIT-TO (PUBYEAR,2020) OR LIMIT-TO (PUBYEAR,2019) OR LIMIT-TO (PUBYEAR,2018) OR LIMIT-TO (PUBYEAR,2017) OR LIMIT-TO (PUBYEAR,2016) OR LIMIT-TO (PUBYEAR,2015) OR LIMIT-TO (PUBYEAR,2014) OR LIMIT-TO (PUBYEAR,2013) OR LIMIT-TO (PUBYEAR,2012) OR LIMIT-TO (PUBYEAR,2011) OR LIMIT-TO (PUBYEAR,2010) OR LIMIT-TO (PUBYEAR,2009) OR LIMIT-TO (PUBYEAR,2008) OR LIMIT-TO (PUBYEAR,2007) OR LIMIT-TO (PUBYEAR,2006)) AND (LIMIT-TO (LANGUAGE,"English") OR LIMIT-TO (LANGUAGE,"Undefined")) **2247**

TITLE-ABS-KEY(Diabet* OR diabetes mellitus OR DM OR IDDM OR NIDDM OR T1DM OR T2DM) AND TITLE-ABS-KEY (impairment* OR "functional loss*" OR weak* OR defic* OR disab* OR disorder* OR dysfunction OR function* OR frail* OR "function* limitation*" OR restriction* OR handicap* OR "activity limitation*" OR "participation restriction*") AND ALL ("South Africa*") AND (LIMIT-TO (PUBYEAR,2021) OR LIMIT-TO (PUBYEAR,2020) OR LIMIT-TO (PUBYEAR,2019) OR LIMIT-TO (PUBYEAR,2018) OR LIMIT-TO (PUBYEAR,2017) OR LIMIT-TO (PUBYEAR,2016) OR LIMIT-TO (PUBYEAR,2015) OR LIMIT-TO (PUBYEAR,2014) OR LIMIT-TO (PUBYEAR,2013) OR LIMIT-TO (PUBYEAR,2012) OR LIMIT-TO (PUBYEAR,2011) OR LIMIT-TO (PUBYEAR,2010) OR LIMIT-TO (PUBYEAR,2009) OR LIMIT-TO (PUBYEAR,2008) OR LIMIT-TO (PUBYEAR,2007) OR LIMIT-TO (PUBYEAR,2006)) AND (LIMIT-TO (AFFILCOUNTRY,"South Africa") OR LIMIT-TO (AFFILCOUNTRY,"Undefined")) AND (LIMIT-TO (LANGUAGE,"English") OR LIMIT-TO (LANGUAGE,"Undefined")) **1445**

TITLE-ABS-KEY (burns) AND TITLE-ABS-KEY (impairment* OR "functional loss*" OR weak* OR defic* OR disab* OR disorder* OR dysfunction OR function* OR frail* OR "function* limitation*" OR restriction* OR handicap* OR "activity limitation*" OR "participation restriction*") AND ALL ("South Africa*") AND (LIMIT-TO (PUBYEAR , 2021) OR LIMIT-TO (PUBYEAR , 2020) OR LIMIT-TO (PUBYEAR , 2019) OR LIMIT-TO (PUBYEAR , 2018) OR LIMIT-TO (PUBYEAR , 2017) OR LIMIT-TO (PUBYEAR , 2016) OR LIMIT-TO (PUBYEAR , 2015) OR LIMIT-TO (PUBYEAR , 2014) OR LIMIT-TO (PUBYEAR , 2013) OR LIMIT-TO (PUBYEAR , 2012) OR LIMIT-TO (PUBYEAR , 2011) OR LIMIT-TO (PUBYEAR , 2010) OR LIMIT-TO (PUBYEAR , 2009) OR LIMIT-TO (PUBYEAR , 2008) OR LIMIT-TO (PUBYEAR , 2007) OR LIMIT-TO (PUBYEAR , 2006)) AND (EXCLUDE (SUBJAREA , "AGRI") OR EXCLUDE (SUBJAREA , "ENVI") OR EXCLUDE (SUBJAREA , "EART") OR EXCLUDE (SUBJAREA , "VETE")) AND (LIMIT-TO (AFFILCOUNTRY , "South Africa") AND (LIMIT-TO (LANGUAGE , "English")) **144**

(TITLE-ABS-KEY(Hearing OR deaf* OR ototoxic* OR ear OR presbycusis) AND TITLE-ABS-KEY(Impairment* OR "functional loss*" OR weak* OR defic* OR disab* OR disorder* OR dysfunction OR function* OR frail* OR "function* limitation*" OR restriction* OR handicap* OR "activity limitation*" OR "participation restriction*") AND ALL("South Africa*") AND (LIMIT-TO (AFFILCOUNTRY,"South Africa") AND (LIMIT-TO (LANGUAGE,"English") OR LIMIT-TO (LANGUAGE,"Afrikaans")) AND (LIMIT-TO (PUBYEAR,2021) OR LIMIT-TO (PUBYEAR,2020) OR LIMIT-TO (PUBYEAR,2019) OR LIMIT-TO (PUBYEAR,2018) OR LIMIT-TO (PUBYEAR,2017) OR LIMIT-TO (PUBYEAR,2016) OR LIMIT-TO (PUBYEAR,2015) OR LIMIT-TO (PUBYEAR,2014) OR LIMIT-TO (PUBYEAR,2013) OR LIMIT-TO (PUBYEAR,2012) OR LIMIT-TO (PUBYEAR,2011) OR LIMIT-TO (PUBYEAR,2010) OR LIMIT-TO (PUBYEAR,2009) OR LIMIT-TO (PUBYEAR,2008) OR LIMIT-TO (PUBYEAR,2007) OR LIMIT-TO (PUBYEAR,2006)) **1226**

TITLE-ABS-KEY(Fracture* OR disloc* OR sublux*) AND TITLE-ABS-KEY(Impairment* OR "functional loss*" OR weak* OR defic* OR disab* OR disorder* OR dysfunction OR function* OR frail* OR "function* limitation*" OR restriction* OR handicap* OR "activity limitation*" OR "participation restriction*") AND ALL("South Africa*") AND (LIMIT-TO (PUBYEAR,2021) OR LIMIT-TO (PUBYEAR,2020) OR LIMIT-TO (PUBYEAR,2019) OR LIMIT-TO (PUBYEAR,2018) OR LIMIT-TO (PUBYEAR,2017) OR LIMIT-TO (PUBYEAR,2016) OR LIMIT-TO (PUBYEAR,2015) OR LIMIT-TO (PUBYEAR,2014) OR LIMIT-TO (PUBYEAR,2013) OR LIMIT-TO (PUBYEAR,2012) OR LIMIT-TO (PUBYEAR,2011) OR LIMIT-TO (PUBYEAR,2010) OR LIMIT-TO (PUBYEAR,2009) OR LIMIT-TO (PUBYEAR,2008) OR LIMIT-TO (PUBYEAR,2007) OR LIMIT-TO (PUBYEAR,2006)) AND (LIMIT-TO (AFFILCOUNTRY,"South Africa") OR LIMIT-TO (AFFILCOUNTRY,"Undefined")) AND (LIMIT-TO (LANGUAGE,"English") OR LIMIT-TO (

LANGUAGE,"Afrikaans")) AND (EXCLUDE (SUBJAREA,"ENGI") OR EXCLUDE (SUBJAREA,"MATE") OR EXCLUDE (SUBJAREA,"EART") OR EXCLUDE (SUBJAREA,"PHYS") OR EXCLUDE (SUBJAREA,"ENVI") OR EXCLUDE (SUBJAREA,"AGRI") OR EXCLUDE (SUBJAREA,"VETE") OR EXCLUDE (SUBJAREA,"CENG") OR EXCLUDE (SUBJAREA,"COMP") OR EXCLUDE (SUBJAREA,"BUSI") OR EXCLUDE (SUBJAREA,"ECON")) **355**

TITLE-ABS-KEY ("reumatoid arthritis" OR "rheumatoid arthritis" OR "rheumatic arthritis" OR "rheumarthritis") AND TITLE-ABS-KEY (impairment* OR "functional loss*" OR weak* OR defic* OR disab* OR disorder* OR dysfunction OR function* OR frail* OR "function* limitation*" OR restriction* OR handicap* OR "activity limitation*" OR "participation restriction*") AND ALL ("South Africa*") AND (LIMIT-TO (PUBYEAR,2021) OR LIMIT-TO (PUBYEAR,2020) OR LIMIT-TO (PUBYEAR,2019) OR LIMIT-TO (PUBYEAR,2018) OR LIMIT-TO (PUBYEAR,2017) OR LIMIT-TO (PUBYEAR,2016) OR LIMIT-TO (PUBYEAR,2015) OR LIMIT-TO (PUBYEAR,2014) OR LIMIT-TO (PUBYEAR,2013) OR LIMIT-TO (PUBYEAR,2012) OR LIMIT-TO (PUBYEAR,2011) OR LIMIT-TO (PUBYEAR,2010) OR LIMIT-TO (PUBYEAR,2009) OR LIMIT-TO (PUBYEAR,2008) OR LIMIT-TO (PUBYEAR,2007) OR LIMIT-TO (PUBYEAR,2006)) AND (LIMIT-TO (AFFILCOUNTRY,"South Africa") OR LIMIT-TO (AFFILCOUNTRY,"Undefined")) AND (LIMIT-TO (LANGUAGE,"English") OR LIMIT-TO (LANGUAGE,"Undefined")) **363**

TITLE-ABS-KEY (gout*) AND TITLE-ABS-KEY (impairment* OR "functional loss*" OR weak* OR defic* OR disab* OR disorder* OR dysfunction OR function* OR frail* OR "function* limitation*" OR restriction* OR handicap* OR "activity limitation*" OR "participation restriction*") AND ALL ("South Africa*") AND (LIMIT-TO (PUBYEAR,2021) OR LIMIT-TO (PUBYEAR,2020) OR LIMIT-TO (PUBYEAR,2019) OR LIMIT-TO (PUBYEAR,2018) OR LIMIT-TO (PUBYEAR,2017) OR LIMIT-TO (PUBYEAR,2016) OR LIMIT-TO (PUBYEAR,2015) OR LIMIT-TO (PUBYEAR,2014) OR LIMIT-TO (PUBYEAR,2013) OR LIMIT-TO (PUBYEAR,2012) OR LIMIT-TO (PUBYEAR,2011) OR LIMIT-TO (PUBYEAR,2010) OR LIMIT-TO (PUBYEAR,2009) OR LIMIT-TO (PUBYEAR,2008) OR LIMIT-TO (PUBYEAR,2007) OR LIMIT-TO (PUBYEAR,2006)) AND (LIMIT-TO (AFFILCOUNTRY,"South Africa") OR LIMIT-TO (AFFILCOUNTRY,"Undefined")) AND (LIMIT-TO (LANGUAGE,"English") OR LIMIT-TO (LANGUAGE,"Undefined")) **52**

TITLE-ABS-KEY (osteoarthriti* OR osteoarthro* OR gonarthriti* OR gonarthro* OR coxarthriti* OR coxarthro* OR arthros* OR arthrot*) AND TITLE-ABS-KEY (impairment* OR "functional loss*" OR weak* OR defic* OR disab* OR disorder* OR dysfunction OR function* OR frail* OR "function* limitation*" OR restriction* OR handicap* OR "activity limitation*" OR "participation restriction*") AND ALL ("South Africa*") AND (LIMIT-TO (PUBYEAR,2021) OR LIMIT-TO (PUBYEAR,2020) OR LIMIT-TO (PUBYEAR,2019) OR LIMIT-TO (PUBYEAR,2018) OR LIMIT-TO (PUBYEAR,2017) OR LIMIT-TO (PUBYEAR,2016) OR LIMIT-TO (PUBYEAR,2015) OR LIMIT-TO (PUBYEAR,2014) OR LIMIT-TO (PUBYEAR,2013) OR LIMIT-TO (PUBYEAR,2012) OR LIMIT-TO (PUBYEAR,2011) OR LIMIT-TO (PUBYEAR,2010) OR LIMIT-TO (PUBYEAR,2009) OR LIMIT-TO (PUBYEAR,2008) OR LIMIT-TO (PUBYEAR,2007) OR LIMIT-TO (PUBYEAR,2006)) AND (LIMIT-TO (AFFILCOUNTRY,"South Africa") OR LIMIT-TO (AFFILCOUNTRY,"Undefined")) AND (LIMIT-TO (LANGUAGE,"English") OR LIMIT-TO (LANGUAGE,"Undefined")) **154**

TITLE-ABS-KEY ("neck pain*" OR "neck ache*" OR neckache* OR "cervical pain*" OR cervicodynia* OR cervicalgia* OR cervicogenic OR whiplash OR torticollis) AND TITLE-ABS-KEY (impairment* OR "functional loss*" OR weak* OR defic* OR disab* OR disorder* OR dysfunction OR function* OR frail* OR "function* limitation*" OR restriction* OR handicap* OR "activity limitation*" OR "participation restriction*") AND ALL ("South Africa*") AND (LIMIT-TO (PUBYEAR,2021) OR LIMIT-TO (PUBYEAR,2020) OR LIMIT-TO (PUBYEAR,2019) OR LIMIT-TO (PUBYEAR,2018) OR LIMIT-TO (PUBYEAR,2017) OR LIMIT-TO (PUBYEAR,2016) OR LIMIT-TO (PUBYEAR,2015) OR LIMIT-TO (PUBYEAR,2014) OR LIMIT-TO (PUBYEAR,2013) OR LIMIT-TO (PUBYEAR,2012) OR LIMIT-TO (PUBYEAR,2011) OR LIMIT-TO (PUBYEAR,2010) OR LIMIT-TO (PUBYEAR,2009) OR LIMIT-TO (PUBYEAR,2008) OR LIMIT-TO (PUBYEAR,2007) OR LIMIT-TO (PUBYEAR,2006)) AND (LIMIT-TO (AFFILCOUNTRY,"South Africa") OR LIMIT-TO (AFFILCOUNTRY,"Undefined")) AND (LIMIT-TO (

LANGUAGE,"English") OR LIMIT-TO (LANGUAGE,"Undefined")) **68**

TITLE-ABS-KEY ("Low back pain" OR backache OR lumbago) AND TITLE-ABS-KEY (impairment* OR "functional loss*" OR weak* OR defic* OR disab* OR disorder* OR dysfunction OR function* OR frail* OR "function* limitation*" OR restriction* OR handicap* OR "activity limitation*" OR "participation restriction*") AND ALL ("South Africa*") AND (LIMIT-TO (PUBYEAR,2021) OR LIMIT-TO (PUBYEAR,2020) OR LIMIT-TO (PUBYEAR,2019) OR LIMIT-TO (PUBYEAR,2018) OR LIMIT-TO (PUBYEAR,2017) OR LIMIT-TO (PUBYEAR,2016) OR LIMIT-TO (PUBYEAR,2015) OR LIMIT-TO (PUBYEAR,2014) OR LIMIT-TO (PUBYEAR,2013) OR LIMIT-TO (PUBYEAR,2012) OR LIMIT-TO (PUBYEAR,2011) OR LIMIT-TO (PUBYEAR,2010) OR LIMIT-TO (PUBYEAR,2009) OR LIMIT-TO (PUBYEAR,2008) OR LIMIT-TO (PUBYEAR,2007) OR LIMIT-TO (PUBYEAR,2006)) AND (LIMIT-TO (AFFILCOUNTRY,"South Africa") OR LIMIT-TO (AFFILCOUNTRY,"Undefined")) AND (LIMIT-TO (LANGUAGE,"English") OR LIMIT-TO (LANGUAGE,"Undefined")) **217**

TITLE-ABS (Headache* OR migraine* OR cephalgia* OR cephalalgi*) AND TITLE-ABS-KEY (impairment* OR "functional loss*" OR weak* OR defic* OR disab* OR disorder* OR dysfunction OR function* OR frail* OR "function* limitation*" OR restriction* OR handicap* OR "activity limitation*" OR "participation restriction*") AND ALL ("South Africa*") AND (LIMIT-TO (AFFILCOUNTRY,"South Africa") OR LIMIT-TO (AFFILCOUNTRY,"Undefined")) AND (LIMIT-TO (PUBYEAR,2021) OR LIMIT-TO (PUBYEAR,2020) OR LIMIT-TO (PUBYEAR,2019) OR LIMIT-TO (PUBYEAR,2018) OR LIMIT-TO (PUBYEAR,2017) OR LIMIT-TO (PUBYEAR,2016) OR LIMIT-TO (PUBYEAR,2015) OR LIMIT-TO (PUBYEAR,2014) OR LIMIT-TO (PUBYEAR,2013) OR LIMIT-TO (PUBYEAR,2012) OR LIMIT-TO (PUBYEAR,2011) OR LIMIT-TO (PUBYEAR,2010) OR LIMIT-TO (PUBYEAR,2009) OR LIMIT-TO (PUBYEAR,2008) OR LIMIT-TO (PUBYEAR,2007) OR LIMIT-TO (PUBYEAR,2006)) AND (LIMIT-TO (LANGUAGE,"English") OR LIMIT-TO (LANGUAGE,"Undefined")) **173**

Total hits for Scopus **9882**

<u>Web of Science (18/08/2021); updated (20/06/2022)</u>

((AB=(Impairment* OR “functional loss*” OR weak* OR defic* OR disab* OR disorder* OR dysfunction OR function* OR frail* OR “function* limitation*” OR restriction* OR handicap* OR “activity limitation*” OR "participation restriction*")) AND ALL=(South Africa*)) AND AB=(Stroke* OR cerebrovascular accident* OR CVA) and SOUTH AFRICA (Countries/Regions) and English (Languages) 2006-01-01 to 2021-12-31 (Index Date) **326**

((AB=(HIV OR HIV-1 OR HIV-2 OR HIV1 OR HIV2 OR ("human immun*" AND virus) OR HIV-infected OR “HIV infect*” OR ("acquired immun*" AND syndrom*) OR HIV/AIDS)) AND AB=(Impairment* OR “functional loss*” OR weak* OR defic* OR disab* OR disorder* OR dysfunction OR function* OR frail* OR “function* limitation*” OR restriction* OR handicap* OR “activity limitation*” OR “participation restriction*”)) AND ALL=(“South Africa*”) **and** SOUTH AFRICA (Countries/Regions) and English or Afrikaans (Language) 2006-01-01 to 2021-12-31 (Index Date) **2 543**

((AB=(Tubercul* OR TB) AND AB=(Impairment* OR “functional loss*” OR weak* OR defic* OR disab* OR disorder* OR dysfunction OR function* OR frail* OR “function* limitation*” OR restriction* OR handicap* OR “activity limitation*” OR “participation restriction*”) AND ALL=(“South Africa*”) and SOUTH AFRICA (Countries/Regions) and English (Languages) 2006-01-01 to 2021-12-31 (Index Date) **1065**

((AB=(Diabet* OR diabetes mellitus OR DM OR IDDM OR NIDDM OR T1DM OR T2DM OR insulin?depend* OR non insulin?depend* OR glucose intoleran* OR insulin resistan*) AND AB=(Impairment* OR “functional loss*” OR weak* OR defic* OR disab* OR disorder* OR dysfunction OR function* OR frail* OR “function* limitation*” OR restriction* OR handicap* OR “activity limitation*” OR “participation restriction*”) AND ALL=(“South Africa*”) and SOUTH AFRICA (Countries/Regions) and English

(Languages) 2006-01-01 to 2021-12-31 (Index Date) **1020**

((AB=(Burn*) AND AB=(Impairment* OR “functional loss*” OR weak* OR defic* OR disab* OR disorder* OR dysfunction OR function* OR frail* OR “function* limitation*” OR restriction* OR handicap* OR “activity limitation*” OR “participation restriction*”) AND ALL=(“South Africa*”) and SOUTH AFRICA (Countries/Regions) and English (Languages) 2006-01-01 to 2021-12-31 (Index Date) **265**

((AB=(Hearing OR deaf* OR ototoxic* OR ear OR presbycusis) AND AB=(Impairment* OR “functional loss*” OR weak* OR defic* OR disab* OR disorder* OR dysfunction OR function* OR frail* OR “function* limitation*” OR restriction* OR handicap* OR “activity limitation*” OR “participation restriction*”) AND ALL=(“South Africa*”) and SOUTH AFRICA (Countries/Regions) and English or Afrikaans (Languages) 2006-01-01 to 2021-12-31 (Index Date) **412**

((AB=(Fracture* OR disloc* OR sublux*) AND AB=(Impairment* OR “functional loss*” OR weak* OR defic* OR disab* OR disorder* OR dysfunction OR function* OR frail* OR “function* limitation*” OR restriction* OR handicap* OR “activity limitation*” OR “participation restriction*”) AND ALL=(“South Africa*”) and SOUTH AFRICA (Countries/Regions) and English or Afrikaans (Languages) and Geology or Materials Science or Engineering or Geochemistry Geophysics or Metallurgy Metallurgical Engineering or Mining Mineral Processing or Environmental Sciences Ecology or Water Resources or Archaeology or Mineralogy or Meteorology Atmospheric Sciences or Zoology or Plant Sciences or Nuclear Science Technology or Marine Freshwater Biology or Government Law or History or Construction Building Technology or Business Economics or Astronomy Astrophysics or Oceanography or Linguistics or Biodiversity Conservation or Architecture or Energy Fuels or Veterinary Sciences (Exclude – Research Areas)2006-01-01 to 2021-12-31 (Index Date) **156**

((AB=("reumatoid arthritis" OR "rheumatoid arthritis" OR "rheumatic arthritis" OR "rheumarthritis") AND AB=(Impairment* OR “functional loss*” OR weak* OR defic* OR disab* OR disorder* OR dysfunction OR function* OR frail* OR “function* limitation*” OR restriction* OR handicap* OR “activity limitation*” OR “participation restriction*”) AND ALL=(“South Africa*”) and SOUTH AFRICA (Countries/Regions) and English (Languages) 2006-01-01 to 2021-12-31 (Index Date) **91**

((AB=(gout*) AND AB=(Impairment* OR “functional loss*” OR weak* OR defic* OR disab* OR disorder* OR dysfunction OR function* OR frail* OR “function* limitation*” OR restriction* OR handicap* OR “activity limitation*” OR “participation restriction*”) AND ALL=(“South Africa*”) and SOUTH AFRICA (Countries/Regions) and English (Languages) 2006-01-01 to 2021-12-31 (Index Date) **17**

((AB=(Osteoarthriti* OR osteoarthro* OR gonarthriti* OR gonarthro* OR coxarthriti* OR coxarthro* OR arthros* OR arthrot*) AND AB=(Impairment* OR “functional loss*” OR weak* OR defic* OR disab* OR disorder* OR dysfunction OR function* OR frail* OR “function* limitation*” OR restriction* OR handicap* OR “activity limitation*” OR “participation restriction*”) AND ALL=(“South Africa*”) and SOUTH AFRICA (Countries/Regions) and English (Languages) 2006-01-01 to 2021-12-31 (Index Date) **68**

((AB=(“neck pain*” OR “neck ache*” OR neckache* OR “cervical pain*” OR cervicodynia* OR cervicalgia* OR cervicogenic OR whiplash OR torticollis) AND AB=(Impairment* OR “functional loss*” OR weak* OR defic* OR disab* OR disorder* OR dysfunction OR function* OR frail* OR “function* limitation*” OR restriction* OR handicap* OR “activity limitation*” OR “participation restriction*”) AND ALL=(“South Africa*”) and SOUTH AFRICA (Countries/Regions) and English (Languages) 2006-01-01 to 2021-12-31 (Index Date) **28**

((AB=(“Low back pain” OR backache OR “spinal pain” OR lumbago OR coccydynia OR spondylosis OR spondylolisthesis OR stenosis OR disc hernia* OR disc prolapse* OR disc degeneration OR slipped disc OR lumb* pain OR scoliosis) AND AB=(Impairment* OR “functional loss*” OR weak* OR defic* OR disab* OR

disorder* OR dysfunction OR function* OR frail* OR "function* limitation*" OR restriction* OR handicap* OR "activity limitation*" OR "participation restriction*") AND ALL=("South Africa*") and SOUTH AFRICA (Countries/Regions) and English (Languages) 2006-01-01 to 2021-12-31 (Index Date) **111**

((AB=(Headache* OR migraine* OR cephalgia* OR cephalalgi*) AND AB=(Impairment* OR "functional loss*" OR weak* OR defic* OR disab* OR disorder* OR dysfunction OR function* OR frail* OR "function* limitation*" OR restriction* OR handicap* OR "activity limitation*" OR "participation restriction*") AND ALL=("South Africa*") and SOUTH AFRICA (Countries/Regions) and English (Languages) 2006-01-01 to 2021-12-31 (Index Date) **111**

Total hits for Web of Science **6213**

Ebscohost (24/08/2021); updated (24/06/2022)

AB (Stroke* OR cerebrovascular accident* OR CVA OR brain vascular accident* OR apoplexy OR brain infarct* OR ((brain OR cerebr* OR cerebell* OR intracran* OR intracerebral) NEAR/3 (ischemi* OR infarct* OR thrombo* OR emboli* OR occlus* OR hypoci*))) AND AB (Impairment* OR "functional loss*" OR weak* OR defic* OR disab* OR disorder* OR dysfunction OR function* OR frail* OR "function* limitation*" OR restriction* OR handicap* OR "activity limitation*" OR "participation restriction*") AND "South Africa*" Limiters - Scholarly (Peer Reviewed) Journals; Published Date: 20060101-20211231; Expanders - Apply equivalent subjects; Narrow by Language: - English; Narrow by Subject Age: - all adult; Search modes - Boolean/Phrase **38**

AB (HIV OR HIV-1 OR HIV-2 OR HIV1 OR HIV2 OR ("human immun*" AND virus) OR HIV-infected OR "HIV infect*" OR ("acquired immun*" AND syndrom*) OR HIV/AIDS) AND AB (Impairment* OR "functional loss*" OR weak* OR defic* OR disab* OR disorder* OR dysfunction OR function* OR frail* OR "function* limitation*" OR restriction* OR handicap* OR "activity limitation*" OR "participation restriction*") AND "South Africa*" Limiters - Scholarly (Peer Reviewed) Journals; Published Date: 20060101-20211231; Expanders - Apply equivalent subjects; Narrow by Language: - English; Narrow by Subject Age: - all adult; Search modes - Boolean/Phrase **275**

AB (Tubercul* OR TB) AND AB (Impairment* OR "functional loss*" OR weak* OR defic* OR disab* OR disorder* OR dysfunction OR function* OR frail* OR "function* limitation*" OR restriction* OR handicap* OR "activity limitation*" OR "participation restriction*") AND "South Africa*" Limiters - Scholarly (Peer Reviewed) Journals; Published Date: 20060101-20211231; Expanders - Apply equivalent subjects; Narrow by Language: - English; Narrow by Subject Age: - all adult; Search modes - Boolean/Phrase **69**

AB (Diabet* OR diabetes mellitus OR DM OR IDDM OR NIDDM OR T1DM OR T2DM OR insulin?depend* OR non insulin?depend* OR glucose intoleran* OR insulin resistan*) AND AB (Impairment* OR "functional loss*" OR weak* OR defic* OR disab* OR disorder* OR dysfunction OR function* OR frail* OR "function* limitation*" OR restriction* OR handicap* OR "activity limitation*" OR "participation restriction*") AND "South Africa*" Limiters - Scholarly (Peer Reviewed) Journals; Published Date: 20060101-20211231; Expanders - Apply equivalent subjects; Narrow by Language: - English; Narrow by Subject Age: - all adult; Search modes - Boolean/Phrase **46**

AB (burn* NOT burnout) AND AB (Impairment* OR "functional loss*" OR weak* OR defic* OR disab* OR disorder* OR dysfunction OR function* OR frail* OR "function* limitation*" OR restriction* OR handicap* OR "activity limitation*" OR "participation restriction*") AND "South Africa*" Limiters - Scholarly (Peer Reviewed) Journals; Published Date: 20060101-20211231; Expanders - Apply equivalent subjects; Narrow by Language: - English; Narrow by Subject Age: - all adult; Search modes - Boolean/Phrase **8**

AB (hearing OR deaf* OR ototoxic* OR ear OR presbycusis) AND AB (Impairment* OR “functional loss*” OR weak* OR defic* OR disab* OR disorder* OR dysfunction OR function* OR frail* OR “function* limitation*” OR restriction* OR handicap* OR “activity limitation*” OR “participation restriction*”) AND “South Africa*” Limiters - Scholarly (Peer Reviewed) Journals; Published Date: 20060101-20211231; Expanders - Apply equivalent subjects; Narrow by Language: - English; Narrow by Subject Age: - all adult; Search modes - Boolean/Phrase **27**

AB (Fracture* OR disloc* OR sublux*) AND AB (Impairment* OR “functional loss*” OR weak* OR defic* OR disab* OR disorder* OR dysfunction OR function* OR frail* OR “function* limitation*” OR restriction* OR handicap* OR “activity limitation*” OR “participation restriction*”) AND “South Africa*” Limiters - Scholarly (Peer Reviewed) Journals; Published Date: 20060101-20211231; Expanders - Apply equivalent subjects; Narrow by Language: - English; Narrow by Subject Age: - all adult; Search modes - Boolean/Phrase **8**

AB ("rheumatoid arthritis") AND AB (Impairment* OR “functional loss*” OR weak* OR defic* OR disab* OR disorder* OR dysfunction OR function* OR frail* OR “function* limitation*” OR restriction* OR handicap* OR “activity limitation*” OR “participation restriction*”) AND “South Africa*” Limiters - Scholarly (Peer Reviewed) Journals; Published Date: 20060101-20211231; Expanders - Apply equivalent subjects; Search modes - Boolean/Phrase **6**

AB (gout*) AND AB (Impairment* OR “functional loss*” OR weak* OR defic* OR disab* OR disorder* OR dysfunction OR function* OR frail* OR “function* limitation*” OR restriction* OR handicap* OR “activity limitation*” OR “participation restriction*”) AND “South Africa*” Limiters - Scholarly (Peer Reviewed) Journals; Published Date: 20060101-20211231; Expanders - Apply equivalent subjects; Search modes - Boolean/Phrase **1**

AB (Osteoarthriti* OR osteoarthro* OR gonarthriti* OR gonarthro* OR coxarthriti* OR coxarthro* OR arthros* OR arthrot*) AND AB (Impairment* OR “functional loss*” OR weak* OR defic* OR disab* OR disorder* OR dysfunction OR function* OR frail* OR “function* limitation*” OR restriction* OR handicap* OR “activity limitation*” OR “participation restriction*”) AND “South Africa*” Limiters - Scholarly (Peer Reviewed) Journals; Published Date: 20060101-20211231; Expanders - Apply equivalent subjects; Narrow by Language: - English; Narrow by Subject Age: - all adult; Search modes - Boolean/Phrase **8**

AB (“neck pain*” OR “neck ache*” OR neckache* OR “cervical pain*” OR cervicodynia* OR cervicalgia* OR cervicogenic OR whiplash OR torticollis) AND AB (Impairment* OR “functional loss*” OR weak* OR defic* OR disab* OR disorder* OR dysfunction OR function* OR frail* OR “function* limitation*” OR restriction* OR handicap* OR “activity limitation*” OR “participation restriction*”) AND “South Africa*” Limiters - Scholarly (Peer Reviewed) Journals; Published Date: 20060101-20211231; Expanders - Apply equivalent subjects; Narrow by Subject Age: - all adult; Search modes - Boolean/Phrase **5**

AB (“Low back pain” OR backache OR “spinal pain” OR lumbago OR coccydynia OR spondylosis OR spondylolisthesis OR stenosis OR disc hernia* OR disc prolapse* OR disc degeneration OR slipped disc OR lumb* pain OR scoliosis) AND AB (Impairment* OR “functional loss*” OR weak* OR defic* OR disab* OR disorder* OR dysfunction OR function* OR frail* OR “function* limitation*” OR restriction* OR handicap* OR “activity limitation*” OR “participation restriction*”) AND “South Africa*” Limiters - Scholarly (Peer Reviewed) Journals; Published Date: 20060101-20211231; Expanders - Apply equivalent subjects; Narrow by Subject Age: - all adult; Search modes - Boolean/Phrase **9**

AB (Headache* OR migraine* OR cephalgia* OR cephalalgi*) AND AB (Impairment* OR “functional loss*” OR weak* OR defic* OR disab* OR disorder* OR dysfunction OR function* OR frail* OR “function* limitation*” OR restriction* OR handicap* OR “activity limitation*” OR “participation restriction*”) AND “South Africa*” Limiters - Scholarly (Peer Reviewed) Journals; Published Date: 20060101-20211231; Expanders - Apply equivalent subjects; Narrow by Language: - English; Narrow by Subject Age: - all adult; Search modes - Boolean/Phrase **10**

Total hits for Ebscohost **510**

SABINET (30/08/2021); updated 29/06/2022

[[All: impairment*] OR [All: "functional loss*"] OR [All: weak*] OR [All: defic*] OR [All: disab*] OR [All: disorder*] OR [All: dysfunction] OR [All: function*] OR [All: frail*] OR [All: "function* limitation*"] OR [All: restriction*] OR [All: handicap*] OR [All: "activity limitation*"] OR [All: "participation restriction*"]] AND [[Publication Title: stroke*] OR [Publication Title: cerebrovascular]] AND [[Publication Title: accident*] OR [Publication Title: cva]] AND [[All: "south africa*"]] **160**

[[All: impairment*] OR [All: "functional loss*"] OR [All: weak*] OR [All: defic*] OR [All: disab*] OR [All: disorder*] OR [All: dysfunction] OR [All: function*] OR [All: frail*] OR [All: "function* limitation*"] OR [All: restriction*] OR [All: handicap*] OR [All: "activity limitation*"] OR [All: "participation restriction*"]] AND [[Publication Title: hiv] OR [Publication Title: hiv-1] OR [Publication Title: hiv-2] OR [Publication Title: hiv1] OR [Publication Title: hiv2] OR [[Publication Title: "human immun*"] AND [Publication Title: virus]] OR [Publication Title: hiv-infected] OR [Publication Title: "hiv infect*"] OR [[Publication Title: "acquired immun*"] AND [Publication Title: syndrom*]] OR [Publication Title: hiv/aids]] AND [All: "south africa*"] AND [Languages: Afrikaans] AND [Languages: English] AND [Publication Date: (01/01/2006 TO 12/31/2021)] **2109**

[[All: impairment*] OR [All: "functional loss*"] OR [All: weak*] OR [All: defic*] OR [All: disab*] OR [All: disorder*] OR [All: dysfunction] OR [All: function*] OR [All: frail*] OR [All: "function* limitation*"] OR [All: restriction*] OR [All: handicap*] OR [All: "activity limitation*"] OR [All: "participation restriction*"]] AND [[Publication Title: tubercul*] OR [Publication Title: tb]] AND [All: "south africa*"] AND [Languages: English] AND [Languages: Afrikaans] AND [Publication Date: (01/01/2006 TO 12/31/2021)] **502**

[[All: diabet*] OR [All: diabetes]] AND [[All: mellitus] OR [All: dm] OR [All: iddm] OR [All: niddm] OR [All: t1dm] OR [All: t2dm] OR [All: insulin?depend*] OR [All: non]] AND [[All: insulin?depend*] OR [All: glucose]] AND [[All: intoleran*] OR [All: insulin]] AND [All: resistan*] AND [[All: impairment*] OR [All: "functional loss*"] OR [All: weak*] OR [All: defic*] OR [All: disab*] OR [All: disorder*] OR [All: dysfunction] OR [All: function*] OR [All: frail*] OR [All: "function* limitation*"] OR [All: restriction*] OR [All: handicap*] OR [All: "activity limitation*"] OR [All: "participation restriction*"]] AND [All: "south africa*"] AND [Publication Date: (01/01/2006 TO 12/31/2021)] **553**

[Publication Title: burns] AND [[All: impairment*] OR [All: "functional loss*"] OR [All: weak*] OR [All: defic*] OR [All: disab*] OR [All: disorder*] OR [All: dysfunction] OR [All: function*] OR [All: frail*] OR [All: "function* limitation*"] OR [All: restriction*] OR [All: handicap*] OR [All: "activity limitation*"] OR [All: "participation restriction*"]] AND [All: "south africa*"] AND [Publication Date: (01/01/2006 TO 12/31/2021)] **147**

[[Publication Title: hearing] OR [Publication Title: deaf*] OR [Publication Title: ototoxic*] OR [Publication Title: ear] OR [Publication Title: presbycusis]] AND [[All: impairment*] OR [All: "functional loss*"] OR [All: weak*] OR [All: defic*] OR [All: disab*] OR [All: disorder*] OR [All: dysfunction] OR [All: function*] OR [All: frail*] OR [All: "function* limitation*"] OR [All: restriction*] OR [All: handicap*] OR [All: "activity limitation*"] OR [All: "participation restriction*"]] AND [All: "south africa*"] AND [Languages: English] AND [Publication Date: (01/01/2006 TO 12/31/2021)] **262**

[[Publication Title: fracture*] OR [Publication Title: disloc*] OR [Publication Title: sublux*]] AND [[All: impairment*] OR [All: "functional loss*"] OR [All: weak*] OR [All: defic*] OR [All: disab*] OR [All: disorder*] OR [All: dysfunction] OR [All: function*] OR [All: frail*] OR [All: "function* limitation*"] OR [All: restriction*] OR [All: handicap*] OR [All: "activity limitation*"] OR [All: "participation restriction*"]] AND [All: "south africa*"] **210**

[[All: "reumatoid arthritis"] OR [All: "rheumatoid arthritis"] OR [All: "rheumatic arthritis"] OR [All: "rheumarthritis"]] AND [[All: impairment*] OR [All: "functional loss*"] OR [All: weak*] OR [All: defic*] OR [All: disab*] OR [All: disorder*] OR [All: dysfunction] OR [All: function*] OR [All: frail*] OR [All: "function*

limitation*"] OR [All: restriction*] OR [All: handicap*] OR [All: "activity limitation*"] OR [All: "participation restriction*"]] AND [All: "south africa*"] AND [Languages: English; Afrikaans] AND [Publication Date: (01/01/2006 TO 12/31/2021)] **418**

[Publication Title: gout*] AND [[All: impairment*] OR [All: "functional loss*"] OR [All: weak*] OR [All: defic*] OR [All: disab*] OR [All: disorder*] OR [All: dysfunction] OR [All: function*] OR [All: frail*] OR [All: "function* limitation*"] OR [All: restriction*] OR [All: handicap*] OR [All: "activity limitation*"] OR [All: "participation restriction*"]] AND [All: "south africa*"] AND [Publication Date: (01/01/2006 TO 12/31/2021)] **38**

[[Publication Title: osteoarthriti*] OR [Publication Title: osteoarthro*] OR [Publication Title: gonarthriti*] OR [Publication Title: gonarthro*] OR [Publication Title: coxarthriti*] OR [Publication Title: coxarthro*] OR [Publication Title: arthros*] OR [Publication Title: arthrot*]] AND [[All: impairment*] OR [All: "functional loss*"] OR [All: weak*] OR [All: defic*] OR [All: disab*] OR [All: disorder*] OR [All: dysfunction] OR [All: function*] OR [All: frail*] OR [All: "function* limitation*"] OR [All: restriction*] OR [All: handicap*] OR [All: "activity limitation*"] OR [All: "participation restriction*"]] AND [All: "south africa*"] AND [Languages: English; Afrikaans] AND [Publication Date: (01/01/2006 TO 12/31/2021)] **26**

[[Publication Title: "neck pain*"] OR [Publication Title: "neck ache*"] OR [Publication Title: neckache*] OR [Publication Title: "cervical pain*"] OR [Publication Title: cervicodynia*] OR [Publication Title: cervicalgia*] OR [Publication Title: cervicogenic] OR [Publication Title: whiplash] OR [Publication Title: torticollis]] AND [[All: impairment*] OR [All: "functional loss*"] OR [All: weak*] OR [All: defic*] OR [All: disab*] OR [All: disorder*] OR [All: dysfunction] OR [All: function*] OR [All: frail*] OR [All: "function* limitation*"] OR [All: restriction*] OR [All: handicap*] OR [All: "activity limitation*"] OR [All: "participation restriction*"]] AND [All: "south africa*"] AND [Publication Date: (01/01/2006 TO 12/31/2021)] **9**

[[All: "low back pain"] OR [All: backache] OR [All: "spinal pain"] OR [All: lumbago] OR [All: coccydynia] OR [All: spondylosis] OR [All: spondylolisthesis] OR [All: stenosis] OR [All: disc]] AND [[All: hernia*] OR [All: disc]] AND [[All: prolapse*] OR [All: disc]] AND [[All: degeneration] OR [All: slipped]] AND [[All: disc] OR [All: lumb*]] AND [[All: pain] OR [All: scoliosis]] AND [[All: impairment*] OR [All: "functional loss*"] OR [All: weak*] OR [All: defic*] OR [All: disab*] OR [All: disorder*] OR [All: dysfunction] OR [All: function*] OR [All: frail*] OR [All: "function* limitation*"] OR [All: restriction*] OR [All: handicap*] OR [All: "activity limitation*"] OR [All: "participation restriction*"]] AND [All: "south africa*"] AND [Publication Date: (01/01/2006 TO 12/31/2021)] **84**

[[Publication Title: "low back pain"] OR [Publication Title: backache]] AND [[All: impairment*] OR [All: "functional loss*"] OR [All: weak*] OR [All: defic*] OR [All: disab*] OR [All: disorder*] OR [All: dysfunction] OR [All: function*] OR [All: frail*] OR [All: "function* limitation*"] OR [All: restriction*] OR [All: handicap*] OR [All: "activity limitation*"] OR [All: "participation restriction*"]] AND [All: "south africa*"] AND [Publication Date: (01/01/2006 TO 12/31/2021)] **92**

[[Publication Title: headache*] OR [Publication Title: migraine *] OR [Publication Title: cephalgia *] OR [Publication Title: cephalalgi *]] AND [[All: impairment*] OR [All: "functional loss*"] OR [All: weak*] OR [All: defic*] OR [All: disab*] OR [All: disorder*] OR [All: dysfunction] OR [All: function*] OR [All: frail*] OR [All: "function* limitation*"] OR [All: restriction*] OR [All: handicap*] OR [All: "activity limitation*"] OR [All: "participation restriction*"]] AND [All: "south africa*"] AND [Publication Date: (01/01/2006 TO 12/31/2021)] **180**

Total hits for SABINET **4790**
